# Antidepressants and movement disorders: a postmarketing study in the world pharmacovigilance database

**DOI:** 10.1186/s12888-020-02711-z

**Published:** 2020-06-16

**Authors:** Alexis Revet, François Montastruc, Anne Roussin, Jean-Philippe Raynaud, Maryse Lapeyre-Mestre, Thi Thu Ha Nguyen

**Affiliations:** 1 Service de Pharmacologie Médicale et Clinique, Centre de Pharmacovigilance, de Pharmacoépidémiologie et d’Informations sur le Médicament, CHU de Toulouse, Faculté de Médecine, Toulouse, France; 2 UMR 1027, Inserm, Université Toulouse III, Toulouse, France; 3 Service Universitaire de Psychiatrie de l’Enfant et de l’Adolescent, CHU de Toulouse, Hôpital Purpan, Place du Dr Baylac, TSA 40031, 31059 Toulouse cedex 9, France; 4 CIC 1436, CHU de Toulouse, Toulouse, France

**Keywords:** Antidepressants, Movement disorders, Case/non-case study, VigiBase®, Pharmacoepidemiology

## Abstract

**Background:**

Antidepressants-induced movement disorders are rare and imperfectly known adverse drug reactions. The risk may differ between different antidepressants and antidepressants’ classes. The objective of this study was to assess the putative association of each antidepressant and antidepressants’ classes with movement disorders.

**Methods:**

Using VigiBase®, the WHO Pharmacovigilance database, disproportionality of movement disorders’ reporting was assessed among adverse drug reactions related to any antidepressant, from January 1967 to February 2017, through a case/non-case design. The association between nine subtypes of movement disorders (akathisia, bruxism, dystonia, myoclonus, parkinsonism, restless legs syndrome, tardive dyskinesia, tics, tremor) and antidepressants was estimated through the calculation first of crude Reporting Odds Ratio (ROR), then adjusted ROR on four potential confounding factors: age, sex, drugs described as able to induce movement disorders, and drugs used to treat movement disorders.

**Results:**

Out of the 14,270,446 reports included in VigiBase®, 1,027,405 (7.2%) contained at least one antidepressant, among whom 29,253 (2.8%) reported movement disorders. The female/male sex ratio was 2.15 and the mean age 50.9 ± 18.0 years. We found a significant increased ROR for antidepressants in general for all subtypes of movement disorders, with the highest association with bruxism (ROR 10.37, 95% CI 9.62–11.17) and the lowest with tics (ROR 1.49, 95% CI 1.38–1.60). When comparing each of the classes of antidepressants with the others, a significant association was observed for all subtypes of movement disorders except restless legs syndrome with serotonin reuptake inhibitors (SRIs) only. Among antidepressants, mirtazapine, vortioxetine, amoxapine, phenelzine, tryptophan and fluvoxamine were associated with the highest level to movement disorders and citalopram, paroxetine, duloxetine and mirtazapine were the most frequently associated with movement disorders. An association was also found with eight other antidepressants.

**Conclusions:**

A potential harmful association was found between movement disorders and use of the antidepressants mirtazapine, vortioxetine, amoxapine, phenelzine, tryptophan, fluvoxamine, citalopram, paroxetine, duloxetine, bupropion, clomipramine, escitalopram, fluoxetine, mianserin, sertraline, venlafaxine and vilazodone. Clinicians should beware of these adverse effects and monitor early warning signs carefully. However, this observational study must be interpreted as an exploratory analysis, and these results should be refined by future epidemiological studies.

## Background

Antidepressants are one of the most frequently prescribed drug classes in Western countries [1–3]. They have broad therapeutic indications, from depression to anxiety or obsessive-compulsive disorders, but also enuresis, chronic pain or eating disorders. The most important classes of antidepressants are serotonin reuptake inhibitors (SRIs), serotonin-norepinephrine reuptake inhibitors (SNRIs), tricyclic antidepressants (TCAs), and monoamine oxidase inhibitors (MAOIs). Antidepressants act mainly through the monoamine neurotransmitters, serotonin and noradrenaline [4, 5]. They can induce several adverse drug reactions [6], including digestive disorders, sexual dysfunction, fatigue or sleepiness, but also hyponatremia, hepatitis [7], or bleeding.

Movement disorders are clinical syndromes with either an excess or a paucity of voluntary and involuntary movements, unrelated to weakness or spasticity. They include extrapyramidal symptoms (akathisia, tardive dyskinesia, dystonia, and parkinsonism) but also a wide range of disorders, from tremor to tics and bruxism, to name a few. Although not the most frequent adverse drug reactions of antidepressants, antidepressant-induced movement disorders have been described and can lead to severe and disabling conditions [8–10]. Reports of extrapyramidal symptoms associated with antidepressants have been documented for SRIs, SNRIs, and other antidepressants [11]. Nevertheless, there are few studies specifically designed to address this association. A recent observational study found a harmful association between the incidence of parkinsonism or associated extrapyramidal symptoms and use of antidepressants duloxetine, mirtazapine, citalopram, escitalopram, paroxetine, sertraline, venlafaxine, bupropion, and fluoxetine [12]. Furthermore, the heterogeneity of movement disorders and the difficulty to correctly label them is a limitation to the quality of the few existing studies. Lastly, the frequent use of psychoactive comedications prone to also induce movement disorders, such as antipsychotics, mood stabilizers or antiepileptics, in patients receiving antidepressants, makes it difficult to precisely assess the level of imputability [8].

Pharmacovigilance is the science and activities relating to the detection, assessment, understanding and prevention of adverse drug reactions or any other drug-related problems [13]. Pharmacovigilance databases, which contain information about patients suffering from adverse drug reactions and the drugs associated to these adverse drug reactions, are of great use to detect potential drug safety signals, or to investigate specific drug-event associations. Among the different analytical methods developed for these large databases, the disproportionality analysis or case/non-case design, which is based on the case-control study principle, is now a well validated exploratory method [14, 15].

The aim of this study was to identify antidepressants’ classes and antidepressants suspected of inducing different subtypes of movement disorders as adverse drug reactions using a case/non-case approach in a worldwide pharmacovigilance database.

## Methods

### Pharmacoepidemiological study

#### Data source

We used VigiBase®, the World Health Organization (WHO) international database of suspected adverse drug reactions, to identify movement disorders related to antidepressants. Since 1978, the WHO Program for International Drug Monitoring is run by the Uppsala Monitoring Center, located in Sweden, which collect and analyze reports of adverse drug reactions transmitted by over 120 countries [16, 17]. Each report of adverse drug reaction includes the available data concerning the reporting country, the notifier’s type, the patient’s demographic characteristics, the drug(s) used and the characteristics of the adverse drug reactions. Adverse drug reactions are coded according to the Medical Dictionary for Regulatory Activities (MedDRA) [18].

The study period for this analysis was defined from 1 January, 1967 to 1 February, 2017.

Analyses in VigiBase® can be performed either directly within data extractions of Vigibase® or via queries in VigiLyzeTM, an online tool developed to help searches by providing an overview of available data [19].

#### Antidepressants and other medications exposure

We identified all reports mentioning the exposure to one of the 58 antidepressants, coded and classified according to the Anatomical Therapeutic Chemical classification system [20], and present in Vigibase® during the study period: 19 TCAs, 8 SRIs, 8 MAOIs, and 23 “other” antidepressants [see details in Table 1 of the Electronic Supplementary Material (ESM)]. For the comparative analysis between antidepressants and movement disorders, drugs known to induce movement disorders, or drugs used to treat movement disorders were identified according to ATC classification (see details in Tables 2 and 3 of the ESM).

**Table 1:** List of Preferred Terms adapted from Standardised MedDRA queries to identify movement disorders reports in VigiBase®

Subtype of Movement Disorders	Preferred Term
Akathisia	Akathisia
Bruxism	Bruxism
Dystonia	Dystonia Dystonic tremor Oculogyric crisis Opisthotonus Oromandibular dystonia Pleurothotonus Torticollis Trismus
Myoclonus	Eyelid myoclonus Myoclonus
Parkinsonism	Akinesia Bradykinesia Hypokinesia Parkinson’s disease Parkinsonian crisis Parkinsonian gait Parkinsonian rest tremor Parkinsonism Reduced facial expression
Restless legs syndrome	Restless legs syndrome
Tardive dyskinesia	Tardive dyskinesia
Tics	Tic
Tremor	Action tremor Essential tremor Intention tremor Postural tremor Resting tremor Tremor

**Table 2:** Characteristics of all reports for the 58 antidepressant drugs of interest in VigiBase® (*n* = 1,027,405)

Characteristics	N	%
**Age group**		
5-17^a^	20,174	1.96
18–44	252,790	24.60
45–64	281,177	27.37
65–74	94,779	9.23
≥ 75	79,894	7.78
Unknown	298,591	29.06
**Sex group**		
Male	309,665	30.14
Female	665,950	64.82
Unknown	51,790	5.04
**WHO reporting area**		
Africa	3507	0.34
Americas		
United States	594,543	57.87
Canada	44,079	4.29
Other countries	5549	0.54
Asia		
China	9827	0.96
India	8284	0.81
Japan	7722	0.75
Singapore	1012	0.10
South Korea	15,919	1.55
Other countries	5863	0.57
Europe		
France	45,142	4.39
Germany	36,334	3.54
Italy	16,180	1.57
United Kingdom	99,131	9.65
Other countries	97,238	9.46
Oceania		
Australia	30,210	2.94
New Zealand	6865	0.67
**Notifier type**		
Physicians	310,728	30.24
Pharmacists	65,062	6.33
Other health professionals	84,365	8.21
Patients	238,572	23.22
Hospitals	38,922	3.79
Lawyers	13,372	1.30
Drugs companies	6470	0.63
Others	124,913	12.16
Unknown	145,001	14.11
**Serious cases**		
Yes	385,788	37.55
No	300,879	29.29
Unknown	340,738	33.16

*WHO,* World Health Organization ^a^Quality of reports under the age of five was uncertain and they were thus excluded

**Table 3:** Results of the case/non-case analysis to identify an increased reporting risk of movement disorder for each of the 9 movement disorders for all antidepressants compared to all other drugs in VigiBase®

	Case	Non-case	Total	Crude ROR [95% CI]	p
**Akathisia**					
58 antidepressants	2160	1,025,245	1,027,405	3.79 [3.61–3.98]	<.0001
All other drugs in VigiBase®	7359	13,235,682	13,243,041		
Total	9519	14,260,927	14,270,446		
**Bruxism**					
58 antidepressants	1244	1,026,161	1,027,405	10.37 [9.62–11.17]	<.0001
All other drugs in VigiBase®	1548	13,241,493	13,243,041		
Total	2792	14,267,654	14,270,446		
**Dystonia**					
58 antidepressants	5113	1,022,292	1,027,405	2.07 [2.01–2.14]	<.0001
All other drugs in VigiBase®	31,870	13,211,171	13,243,041		
Total	36,983	14,233,463	14,270,446		
**Myoclonus**					
58 antidepressants	1944	1,025,461	1,027,405	4.79 [4.55–5.05]	<.0001
All other drugs in VigiBase®	5237	13,237,804	13,243,041		
Total	7181	14,263,265	14,270,446		
**Parkinsonism**					
58 antidepressants	3695	1,023,710	1,027,405	2.14 [2.07–2.22]	<.0001
All other drugs in VigiBase®	22,289	13,220,752	13,243,041		
Total	25,984	14,244,462	14,270,446		
**Restless legs syndrome**					
58 antidepressants	2430	1,024,975	1,027,405	5.14 [4.90–5.38]	<.0001
All other drugs in VigiBase®	6111	13,236,930	13,243,041		
Total	8541	14,261,905	14,270,446		
**Tardive dyskinesia**					
58 antidepressants	2598	1,024,807	1,027,405	1.55 [1.49–1.61]	<.0001
All other drugs in VigiBase®	21,625	13,221,416	13,243,041		
Total	24,223	14,246,223	14,270,446		
**Tics**					
58 antidepressants	770	1,026,635	1,027,405	1.49 [1.38–1.60]	<.0001
All other drugs in VigiBase®	6667	13,236,374	13,243,041		
Total	7437	14,263,009	14,270,446		
**Tremor**					
58 antidepressants	28,021	999,384	1,027,405	3.06 [3.02–3.10]	<.0001
All other drugs in VigiBase®	120,400	13,122,641	13,243,041		
Total	148,421	14,122,025	14,270,446		

*ROR, reporting odds ration; CI, confident interval*

#### Reports of movement disorders

We used the MedDRA terms to identify reports of movement disorders in VigiBase® [18]. We selected nine subtypes of movement disorders which have been previously described as potentially induced by antidepressants: akathisia, bruxism, dystonia, myoclonus, parkinsonism, restless legs syndrome, tardive dyskinesia, tics, and tremor [8].

The MedDRA dictionary is organized into five hierarchical levels, from the least to the most precise: ‘System Organ Class’, ‘High-Level Groups Terms’, ‘High-Level Terms, ‘Preferred Terms’, and ‘Lowest Level Terms’. In this study, the reports of movement disorders were defined according to the ‘Preferred Terms’ (Table 1). The MedDRA terms were selected by three authors (AR1, FM and MLM), specialized in neuropsychopharmacology and movement disorders.

#### Case/non-case study

The quantitative association of drug-related adverse drug reaction was detected by estimating a measure of disproportionality, expressed as the reporting odds ratio (ROR). The ROR of a combination of interest drug–adverse drug reaction was defined as the ratio between proportions of reports containing the drug of interest in the “case” (reports containing the adverse drug reaction of interest) and in the “non-case” (reports containing other adverse drug reactions) groups [14, 15]. In other words, in the present study the ROR allows to measure the risk of movement disorder reporting among all other adverse drug reactions for all studied antidepressants. This tool is easily reproducible and can be adjusted for potential cofounders in logistic regression models.

### Statistical analyses 

“Cases” were defined as reports of movement disorders and “non-cases” corresponded to the remaining reports of adverse drug reactions in VigiBase®. ROR, which assessed the strength of the association between antidepressants and movement disorders’ occurrence, were given with their 95% confidence interval (CI).

First, we performed a case/non-case analysis to investigate a putative movement disorders association for each of the nine selected movement disorders for all antidepressants in general, which were compared to all the other drugs registered in VigiBase®. Analyses were performed via queries in VigiLyzeTM [19] and results were given as crude RORs.

Second, using the extraction of 1,027,405 reports containing at least one antidepressant, we ranked increased movement disorders reporting according the four classes of antidepressants and the 58 antidepressants. We used adjusted logistic regression models and results were expressed as adjusted RORs (aRORs) on four potential confounding factors: age, sex, drugs described as able to induce movement disorders, and drugs used to treat movement disorders (see details in Tables 2 and 3 of the ESM). We excluded reports with missing values for these factors, reports without detailed adverse drug reactions, and reports containing more than one antidepressant. We also excluded outlying data on patient age.

We conducted sensitivity analyses for the antidepressants most frequently reported with movement disorders among 58 antidepressants. For each of these antidepressants, instead of using the study period in the main analysis, we counted the study period from the year registering its first report in VigiBase® to 1 February, 2017.

In order to minimize the risk of type I error from multiple comparisons, the significance level was adjusted using the Bonferroni’s corrections and was set to 0.001 (alpha = 0.05/58 = 0.000862 which was rounded to 0.001, 58 being the number of comparisons, one for each antidepressants) [21].

All calculations were performed using SAS® software (SAS Institute, Cary, NC, USA).

## Results

### Characteristic of cases

During the study period, out of the 14,270,446 reports included in VigiBase®, 1,027,405 (7.2%) reports contained at least one of the 58 antidepressants. More than half of the reports came from USA (57.9%), followed by the UK (9.6%), France (4.4%), and Germany (3.5%). The highest number of reports (118,526) was found in 2015. The female/male sex ratio was 2.15 and the mean age was 50.9 ± 18.0 years. A majority of cases were “serious” (37.6%). These data are presented in detail in Table 2.

**Fig. 1:**
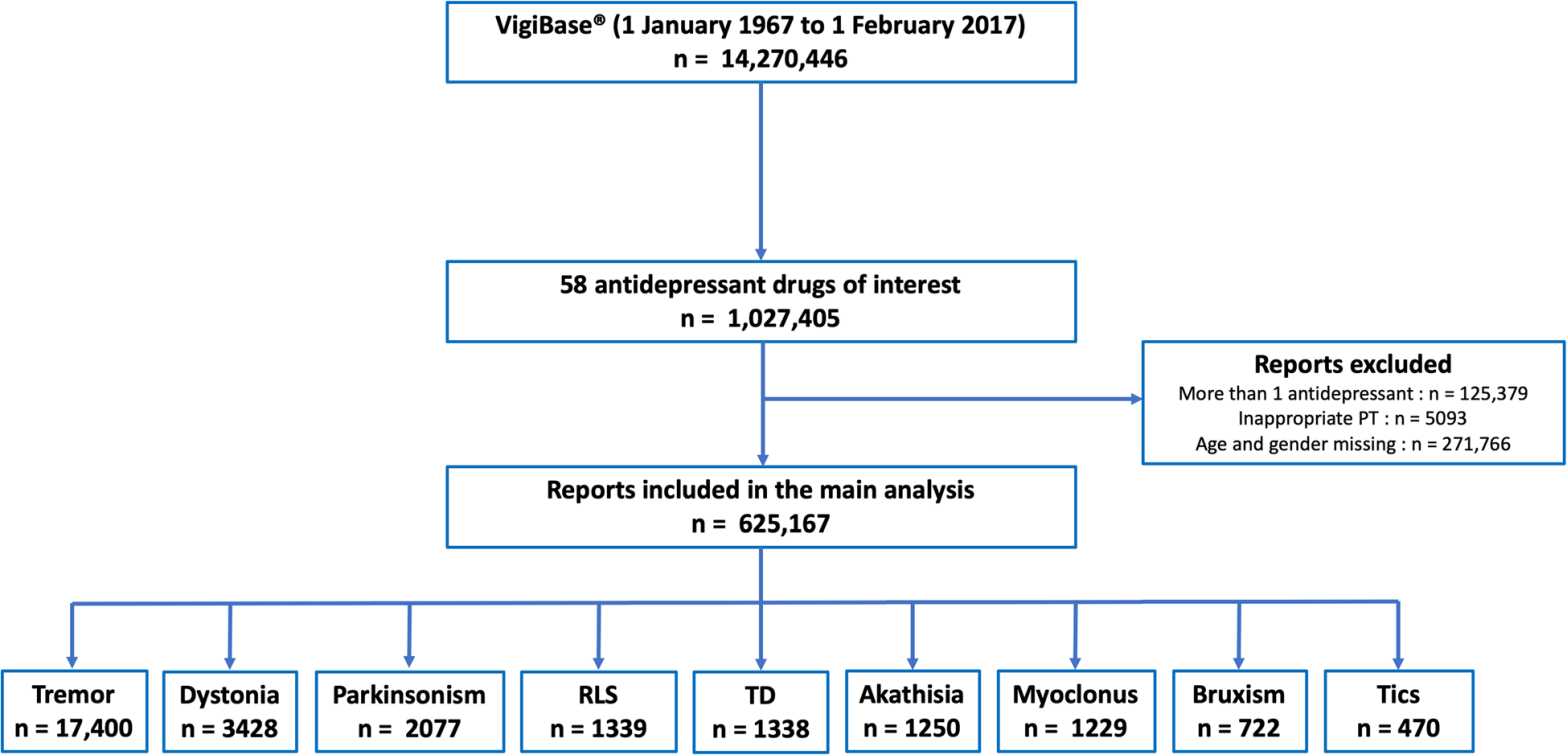
Flowchart of the study protocol. *PT, Preferred Terms; RLS, restless legs syndrme; TD, tardive dyskinesia; n, number of reports*

Among these 1,027,405 reports of ADs, we identified 29,253 (2.8%) reports of movements disorders: 17,400 reports of tremor, 3428 reports of dystonia, 2077 reports of parkinsonism, 1339 reports of restless legs syndrome, 1338 reports of tardive dyskinesia, 1250 reports of akathisia, 1229 reports of myoclonus, 722 reports of bruxism, and 470 reports of tics (Fig. 1). The first movement disorder’s report was reported in 1968 with a TCA. The first report of this adverse drug reaction with MAOIs was in 1969 and in 1982 with SRIs.

### Case/non-case study

#### Comparison with all other drugs

Comparison of antidepressants in general (taken as a whole) with all other drugs in VigiBase® showed a significant increased crude ROR for all subtypes of movement disorders (Table 3): bruxism (ROR 10.37, 95% CI 9.62–11.17); restless legs syndrome (ROR 5.14, 95% CI 4.90–5.38); myoclonus (ROR 4.79, 95% CI 4.55–5.05); akathisia (ROR 3.79, 95% CI 3.61–3.98); tremor (ROR 3.06, 95% CI 3.02–3.10); parkinsonism (ROR 2.14, 95% CI 2.07–2.22); dystonia (ROR 2.07, 95% CI 2.01–2.14); tardive dyskinesia (ROR 1.55, 95% CI 1.49–1.61); and tics (ROR 1.49, 95% CI 1.38–1.60).

#### Comparison between classes of antidepressants and between the different antidepressants

From the 1,027,405 reports containing at least one of the 58 ADs, we finally included 625,167 reports in the adjusted logistic regression model (291,020 for SRIs, 223,561 for “other” antidepressants, 103,139 for TCAs, and 7447 for MAOIs) to perform the comparative analysis below (Fig. 1)

When comparing each of the four classes of antidepressants with the three other classes, we found a significant movement disorders association for all subtypes of movement disorders except restless legs syndrome only for SRIs, but not for TCAs, MAOIs, and “other” antidepressants (Table 4).

**Table 4:** Adjusted reporting odds ratio for the 9 movement disorders between different classes of antidepressants and between the different antidepressants

Antidepressants	aROR^**a**^ (95% CI)								
	Akathisia	Bruxism	Dystonia	Myoclonus	Parkinsonism	RLS	TD	Tics	Tremor
Tricyclic antidepressants	0.40 [0.32–0.49]	0.13 [0.08–0.21]	0.88 [0.80–0.97]	0.87 [0.74–1.02]	0.77 [0.68–0.87]	0.36 [0.29–0.45]	0.80 [0.69–0.93]	0.48 [0.34–0.67]	0.76 [0.73–0.79]
Serotonin reuptake inhibitors	1.50 [1.34–1.68]*	1.56 [1.35–1.82]*	1.66 [1.55–1.78]*	1.25 [1.12–1.40]*	1.24 [1.14–1.35]*	0.82 [0.73–0.91]*	1.21 [1.08–1.34]*	1.20 [1.00–1.44]*	1.20 [1.16–1.23]*
Monoamine oxidase inhibitors	0.51 [0.25–1.02]	0.34 [0.11–1.07]	0.96 [0.70–1.32]	2.54 [1.81–3.56]	1.12 [0.75–1.66]	0.27 [0.10–0.72]	0.79 [0.46–1.37]	0.19 [0.03–1.36]	0.77 [0.66–0.90]
“Other” antidepressants	0.98 [0.88–1.11]	1.14 [0.98–1.33]	0.59 [0.54–0.64]	0.78 [0.69–0.88]	0.92 [0.84–1.01]	1.89 [1.70–2.10]	0.94 [0.84–1.05]	1.17 [0.97–1.41]	0.97 [0.94–1.00]
Agomelatine	1.31 [0.49–3.50]	1.02 [0.26–4.11]	0.34 [0.11–1.05]	1.30 [0.49–3.47]	1.06 [0.44–2.57]	2.11 [1.00–4.44]	0.33 [0.05–2.34]	1.70 [0.42–6.84]	0.45 [0.29–0.69]
Amineptine	**–**	**–**	0.41 [0.10–1.62]	1.16 [0.29–4.64]	1.03 [0.33–3.22]	**–**	**–**	**–**	0.31 [0.15–0.61]
Amitriptyline	0.22 [0.14–0.34]	0.14 [0.07–0.29]	0.65 [0.56–0.76]	0.88 [0.71–1.09]	0.67 [0.56–0.80]	0.44 [0.33–0.58]	0.60 [0.47–0.76]	0.48 [0.29–0.81]	0.64 [0.60–0.69]
Amoxapine	3.22 [1.60–6.48]	**–**	4.43 [3.07–6.39]*	1.26 [0.41–3.93]	2.02 [1.04–3.92]	0.41 [0.06–2.90]	4.42 [2.50–7.84]*	**–**	0.65 [0.43–0.98]
Bifemelane	**–**	**–**	**–**	**–**	8.28 [1.06–64.44]	**–**	**–**	**–**	**–**
Bupropion	0.32 [0.23–0.46]	0.67 [0.48–0.92]	0.38 [0.32–0.46]	0.46 [0.34–0.63]	0.75 [0.61–0.93]	0.67 [0.52–0.86]	0.83 [0.66–1.05]	0.85 [0.60–1.20]	1.35 [1.28–1.42]*
Butriptyline	**–**	**–**	**–**	**–**	**–**	**–**	**–**	**–**	**–**
Citalopram	1.60 [1.39–1.84]*	1.48 [1.22–1.78]*	0.95 [0.85–1.05]	1.31 [1.13–1.52]*	1.21 [1.08–1.36]	0.98 [0.84–1.14]	1.07 [0.92–1.25]	1.00 [0.77–1.30]	0.92 [0.88–0.96]
Clomipramine	0.63 [0.36–1.08]	0.19 [0.05–0.77]	1.33 [1.05–1.69]	2.73 [2.01–3.71]*	1.33 [0.97–1.82]	0.24 [0.09–0.64]	0.72 [0.43–1.22]	0.79 [0.36–1.78]	1.42 [1.27–1.59]*
Desipramine			0.87 [0.52–1.44]	0.86 [0.32–2.29]	0.62 [0.26–1.50]	0.42 [0.10–1.68]	1.24 [0.59–2.60]	0.40 [0.06–2.83]	1.09 [0.86–1.37]
Desvenlafaxine	0.62 [0.30–1.31]	2.01 [1.18–3.41]	0.66 [0.43–1.02]	0.18 [0.05–0.72]	0.64 [0.35–1.20]	1.37 [0.86–2.18]	1.38 [0.86–2.23]	0.23 [0.03–1.66]	1.17 [1.01–1.35]
Dibenzepin	**–**	**–**	**–**	**–**	**–**	**–**	**–**	**–**	1.76 [0.96–3.23]
Dosulepin	0.47 [0.18–1.26]	0.40 [0.10–1.59]	1.18 [0.80–1.72]	0.32 [0.10–0.98]	0.61 [0.34–1.11]	0.31 [0.10–0.97]	0.88 [0.46–1.70]		0.75 [0.61–0.91]
Doxepin	0.47 [0.22–0.99]	0.12 [0.02–0.88]	0.79 [0.55–1.14]	0.37 [0.17–0.84]	0.82 [0.82–1.22]	0.49 [0.25–0.99]	1.11 [0.72–1.73]	0.61 [0.20–1.89]	0.68 [0.57–0.80]
Duloxetine	1.15 [0.92–1.43]	1.83 [1.46–2.31]*	0.58 [0.48–0.69]	0.74 [0.58–0.96]	0.84 [0.70–1.02]	1.88 [1.61–2.20]*	0.94 [0.76–1.18]	1.26 [0.89–1.80]	1.16 [1.10–1.23]*
Escitalopram	2.02 [1.69–2.42]*	1.62 [1.25–2.10]*	0.81 [0.69–0.95]	0.91 [0.71–1.17]	1.16 [0.97–1.38]	1.11 [0.89–1.38]	1.18 [0.96–1.47]	1.14 [0.79–1.64]	0.97 [0.91–1.04]
Etoperidone	**–**	**–**	**–**	**–**	**–**	**–**	**–**	**–**	6.88 [2.01–23.50]
Fluoxetine	1.02 [0.86–1.20]	0.66 [0.51–0.85]	1.51 [1.39–1.65]*	1.02 [0.85–1.22]	0.94 [0.81–1.09]	0.64 [0.52–0.79]	1.22 [1.04–1.43]	0.94 [0.72–1.24]	0.96 [0.92–1.01]
Fluvoxamine	1.61 [1.13–2.29]	1.28 [0.74–2.22]	1.48 [1.18–1.86]*	1.81 [1.25–2.64]	1.05 [0.73–1.52]	0.68 [0.38–1.23]	0.87 [0.52–1.44]	1.12 [0.58–2.17]	1.73 [1.56–1.92]*
*Hypericum perforatum*	**–**	**–**	0.26 [0.09–0.82]	0.72 [0.23–2.24]	0.70 [0.26–1.86]	0.71 [0.23–2.20]	0.25 [0.04–1.77]	**–**	0.36 [0.24–0.54]
Imipramine	0.58 [0.34–1.01]	0.08 [0.01–0.58]	1.17 [0.93–1.48]	0.77 [0.46–1.28]	0.65 [0.43–0.97]	0.50 [0.27–0.94]	1.11 [0.75–1.65]	0.76 [0.36–1.61]	0.95 [0.84–1.07]
Iprindole	**–**	**–**	2.01 [0.28–14.52]	**–**	**–**	**–**	**–**	**–**	**–**
Iproclozide	**–**	**–**	**–**	**–**	**–**	**–**	**–**	**–**	**–**
Iproniazide	**–**	**–**	**–**	4.92 [0.69–35.06]	4.61 [1.10–19.37]	**–**	**–**	**–**	0.69 [0.17–2.79]
Isocarboxazid	**–**	**–**	**–**	3.31 [0.46–23.59]	**–**	**–**	2.51 [0.35–18.01]	**–**	0.87 [0.32–2.34]
Lofepramine	0.63 [0.24–1.68]		0.61 [0.34–1.11]	0.31 [0.08–1.25]	0.72 [0.37–1.39]		0.70 [0.29–1.69]		0.70 [0.55–0.89]
Maprotiline	1.23 [0.61–2.46]	**–**	0.49 [0.25–0.93]	0.96 [0.43–2.14]	1.66 [1.05–2.61]	0.15 [0.02–1.08]	0.42 [0.14–1.32]	**–**	0.75 [0.59–0.95]
Medifoxamine	**–**	**–**	**–**	**–**	**–**	**–**	**–**	**–**	1.14 [0.28–4.65]
Melitracen	0.95 [0.13–6.80]	**–**	2.14 [0.95–4.79]	**–**	3.87 [2.00–7.50]	**–**	4.85 [2.17–10.87]	6.06 [1.51–24.42]	1.71 [1.19–2.45]
Mianserin	2.49 [1.66–3.74]*	**–**	0.64 [0.39–1.05]	1.52 [0.96–2.43]	0.75 [0.48–1.16]	0.95 [0.52–1.72]	0.30 [0.11–0.81]	**–**	0.49 [0.39–0.61]
Milnacipran	0.71 [0.23–2.22]	0.35 [0.05–2.50]	0.33 [0.13–0.89]	0.41 [0.10–1.63]	0.67 [0.28–1.60]	0.16 [0.02–1.12]	0.38 [0.10–1.52]	**–**	0.90 [0.70–1.15]
Minaprine	**–**	**–**	2.12 [0.29–15.30]	**–**	2.77 [0.38–20.05]	**–**	**–**	**–**	0.77 [0.19–3.14]
Mirtazapine	2.55 [2.06–3.14]*	0.42 [0.22–0.82]	0.94 [0.76–1.15]	1.61 [1.26–2.06]*	1.24 [1.02–1.50]	5.24 [4.51–6.10]*	1.11 [0.84–1.47]	0.50 [0.24–1.06]	0.71 [0.64–0.78]
Moclobemide	0.83 [0.35–2.00]	0.29 [0.04–2.04]	1.13 [0.71–1.80]	0.83 [0.34–1.99]	1.09 [0.60–1.97]	0.49 [0.16–1.54]	0.31 [0.08–1.23]	**–**	0.72 [0.56–0.93]
Nefazodone	0.88 [0.47–1.65]	**–**	0.73 [0.48–1.11]	0.28 [0.09–0.88]	1.09 [0.66–1.78]	0.37 [0.14–0.99]	0.36 [0.13–0.96]	0.49 [0.12–1.95]	0.64 [0.53–0.78]
Nialamide	**–**	**–**	**–**	**–**	**–**	**–**	**–**	**–**	**–**
Nomifensine	**–**	**–**	0.58 [0.22–1.56]	**–**	**–**	**–**	**–**	**–**	0.53 [0.34–0.83]
Nortriptyline	0.48 [0.24–0.97]	0.33 [0.11–1.01]	0.98 [0.72–1.33]	0.34 [0.15–0.77]	0.63 [0.41–0.98]	0.31 [0.14–0.68]	1.03 [0.67–1.59]	0.36 [0.09–1.44]	0.84 [0.73–0.96]
Opipramol	**–**	**–**	0.66 [0.21–2.04]	0.53 [0.07–3.73]	0.61 [0.15–2.43]	0.52 [0.07–3.67]	0.53 [0.07–3.74]	**–**	0.72 [0.72–1.13]
Oxaflozane	**–**	**–**	**–**	**–**	**–**	**–**	**–**	**–**	**–**
Oxitriptan	**–**	**–**	0.86 [0.12–6.16]	3.02 [0.42–21.71]	3.68 [0.91–14.97]	2.69 [0.38–19.19]	**–**	**–**	1.00 [0.41–2.43]
Paroxetine	1.49 [1.27–1.76]*	1.43 [1.15–1.78]	1.87 [1.70–2.05]*	1.43 [1.21–1.69]*	1.16 [1.01–1.34]	1.18 [0.99–1.41]	1.22 [1.02–1.45]	1.20 [0.90–1.59]	1.52 [1.45–1.58]*
Phenelzine	0.57 [0.18–1.76]	**–**	0.85 [0.48–1.50]	4.85 [3.14–7.49]*	1.28 [0.64–2.58]	**–**	0.37 [0.09–1.50]	**–**	0.83 [0.64–1.08]
Protriptyline	**–**	**–**	1.45 [0.54–3.90]	1.15 [0.16–8.20]	**–**	1.08 [0.15–7.71]	**–**	**–**	0.81 [0.45–1.47]
Quinupramine	**–**	**–**	**–**	26.74 [3.57–200.29]	**–**	**–**	**–**	**–**	1.94 [0.26–14.27]
Reboxetine	1.95 [0.87–4.36]	0.58 [0.08–4.09]	0.70 [0.31–1.55]	0.36 [0.05–2.52]	0.69 [0.22–2.14]	0.36 [0.05–2.57]	0.36 [0.05–2.53]		0.62 [0.42–0.92]
Sertraline	0.85 [0.71–1.02]	1.63 [1.35–1.98]*	1.02 [0.92–1.13]	0.74 [0.61–0.91]	1.23 [1.08–1.39]	0.80 [0.66–0.97]	1.03 [0.87–1.22]	1.33 [1.04–1.71]	1.08 [1.03–1.14]*
Tianeptine	0.35 [0.05–2.45]	**–**	0.28 [0.07–1.12]	1.58 [0.71–3.53]	0.75 [0.34–1.68]	0.26 [0.04–1.86]	**–**	**–**	0.51 [0.35–0.75]
Toloxatone	**–**	**–**	**–**	5.86 [0.82–42.07]	**–**	**–**	**–**	**–**	0.42 [0.06–3.04]
Tranylcypromine	**–**	1.11 [0.28–4.47]	1.06 [0.55–2.04]	2.11 [0.94–4.71]	0.87 [0.32–2.32]	0.31 [0.04–2.18]	2.20 [1.09–4.42]	0.94 [0.13–6.69]	0.83 [0.60–1.16]
Trazodone	1.08 [0.80–1.46]	0.44 [0.23–0.85]	1.01 [0.83–1.24]	0.48 [0.31–0.74]	1.03 [0.83–1.28]	1.01 [0.75–1.36]	1.31 [1.01–1.69]	0.58 [0.29–1.17]	0.73 [0.66–0.81]
Trimipramine	1.06 [0.44–2.55]	0.40 [0.06–2.81]	1.57 [1.00–2.47]	0.63 [0.20–1.95]	0.52 [0.22–1.25]	0.81 [0.30–2.15]	1.30 [0.62–2.74]	**–**	0.93 [0.72–1.20]
Tryptophan	0.10 [0.03–0.41]	0.08 [0.01–0.54]	0.07 [0.03–0.19]	0.05 [0.01–0.28]	0.05 [0.01–0.20]	0.04 [0.01–0.30]	0.09 [0.02–0.35]	8.13 [6.06–10.92]*	0.36 [0.30–0.43]
Venlafaxine	0.98 [0.80–1.19]	1.98 [1.63–2.42]*	1.02 [0.90–1.15]	1.31 [1.09–1.57]	1.22 [1.06–1.42]	1.25 [1.06–1.49]	1.35 [1.14–1.59]*	0.81 [0.57–1.16]	1.07 [1.02–1.13]
Vilazodone	0.75 [0.24–2.32]	2.04 [0.85–4.92]	0.18 [0.05–0.72]	**–**	1.48 [0.74–2.97]	3.87 [2.40–6.26]*	1.35 [0.61–3.02]	0.64 [0.09–4.54]	1.41 [1.13–1.76]
Viloxazine	**–**	**–**	**–**	4.66 [1.93–11.28]	1.57 [0.58–4.22]	**–**	**–**	**–**	0.43 [0.19–0.95]
Vortioxetine	2.17 [1.03–4.57]	4.71 [2.52–8.80]*	0.51 [0.21–1.23]	0.31 [0.04–2.15]	0.47 [0.12–1.87]	0.82 [0.26–2.55]	0.87 [0.28–2.71]	2.23 [0.72–6.95]	0.59 [0.41–0.85]
Zimeldine	**–**	**–**	0.47 [0.12–1.90]	1.22 [0.30–4.87]	**–**	**–**	**–**	**–**	0.47 [0.26–0.82]

*CI confidence interval, aROR adjusted reporting odd ratio; RLS, restless legs syndrome; TD, tardive dyskinesia*

^a^Adjusted ROR were calculated in adjusted univariate logistic regression analysis, with adjustment for age, gender, drugs described as able to induce movement disorders, and drugs used to treat movement disorders

* Significant association was defined as adjusted ROR > 1 with α threshold of 0.001, and the number of cases being at least 10

Among individual antidepressants, we found a significant association for all subtypes of movement disorders except parkinsonism. Mirtazapine, vortioxetine, amoxapine, phenelzine, tryptophan, and fluvoxamine were the antidepressants associated with the highest aROR value to movement disorders. Citalopram, paroxetine, duloxetine, and mirtazapine were the antidepressants most frequently associated with movement disorders. An association was also found with bupropion, clomipramine, escitalopram, fluoxetine, mianserin, sertraline, venlafaxine, and vilazodone. Table 4 shows data about associations between the subtypes of movement disorders and the different antidepressants (see details in Tables 4.a. to 4.i. of the ESM).

The sensitivity analyses restricting the study period for the antidepressants most frequently reported with movement disorders as compared with other antidepressants showed overall persistent associations (see details in Table 5 of the ESM).

## Discussion

### Key findings

The most frequently notified movement disorder after antidepressant exposure was tremor and the least frequently notified was tics. When comparing antidepressants taken as a whole with all other drugs in VigiBase®, we found a significant increased ROR for all subtypes of movement disorders, with the highest association with bruxism and the lowest with tics. When comparing each of the four classes of antidepressants with the three other classes, we found a significant movement disorders association for all subtypes of movement disorders only for SRIs. Among antidepressants, six “other” antidepressants (mirtazapine, vortioxetine, amoxapine, phenelzine, tryptophan, and fluvoxamine) were associated with the highest aROR to movement disorders and two SRIs (citalopram and paroxetine) and two “other” antidepressants (duloxetine and mirtazapine) were the most frequently associated with movement disorders. An association was also found with one TCA (clomipramine), three SRIs (escitalopram, fluoxetine and sertraline), and four “other” antidepressants (bupropion, mianserin, venlafaxine, and vilazodone).

### Discussion of research findings

Although this type of pharmacovigilance study cannot lead to a precise evaluation of adverse drug reactions’ frequencies, our results tend to confirm the fact that antidepressant-induced movements disorders are rare adverse drug reactions (only 2.8% of reports containing at least one antidepressant). In a recent review [8], Fenelon highlighted the lack of precise data concerning the frequency of these adverse drug reactions, which he related to the following reasons: “a) the rarity of systematic prospective studies properly designed to detect movement disorders; b) the use of ill-defined terms, such as “extrapyramidal symptoms” in the older medical literature; c) finally, the fact that a number of patients receiving antidepressants also receive other psychoactive drugs that may also generate movement disorders (e.g., neuroleptics, lithium), so that the imputability may be difficult to establish.” However, the very high lifetime prevalence of depression [22] balances the rarity of these adverse drug reactions, which clinicians should not ignore.

Although our study aimed to identify antidepressants suspected of inducing different movement disorders using a case/non-case approach and did not focus on the identification of factors associated with a higher risk of association, such as sex or age, these data would be important for guiding clinical decision. Some studies have suggested that parkinsonism on SRIs would be more frequent in older age (65 years and older) and in female [23, 24]. Contradictory results were obtained concerning the association between restless legs syndrome and gender [25]. To date, little information is available on the subject and future studies should precise these sociodemographic risk factors.

One cluster of antidepressants-induced movement disorder are extrapyramidal symptoms which include akathisia, tardive dyskinesia, dystonia, and parkinsonism. Although the precise mechanism of the association between extrapyramidal symptoms and antidepressants is not precisely known, it has been proposed that the increase in the availability of serotonin could indirectly inhibit dopamine release in the striatum by increasing the stimulation of 5-HT_2_ receptors [11, 26]. Within this theoretical framework, the variation in affinity for 5-HT_2_ receptors between antidepressants may explain the differences in the frequency and the intensity of extrapyramidal symptoms seen in patients. A report using data from a multicenter drug-surveillance program on 15 patients between 1994 and 2016 [27], found that extrapyramidal symptoms frequently occurred with SRIs treatment alone (7/15 cases) or concomitant SRI treatment (6/15 cases) and were most frequent with escitalopram-treatment (5 cases). The authors found that the most common extrapyramidal symptom was atypical dyskinesia (6/15 cases) followed by akathisia (4/15 cases) and extrapyramidal symptoms occurred at any dosage and equally often in men and women. A recent nested case-control study was conducted using a large health claims database in the United States from June 2006 to December 2015 and found a harmful association between extrapyramidal symptoms and duloxetine, mirtazapine, citalopram, escitalopram, paroxetine, sertraline, venlafaxine, bupropion, and fluoxetine [12]. In our study, citalopram, escitalopram, mirtazapine, and paroxetine were associated with akathisia, fluoxetine and paroxetine were associated with dystonia, and venlafaxine was associated with tardive dyskinesia.

Antidepressant-induced akathisia has been described following treatment with TCAs [28], SRIs, in particular fluoxetine [29], and mirtazapine [30]. A case report in a 22-year-old woman suggested a link between akathisia and the severity of depressive symptoms, in particular suicidal ideation, after an increase in the dose of fluoxetine [29]. Interestingly, a study highlighted the interest of trazodone, an antidepressant with serotoninergic antagonist properties, for the treatment of neuroleptic-induced akathisia [31].

Although dystonia and tardive dyskinesia associated with antidepressants have been described [23, 32], precise data are missing [8]. In our study, amoxapine, a tetracyclic antidepressant, was associated with the highest aROR for these two movement disorders. These associations have been described in several case reports [33–35], with a positive effect of anticholinergic agents on patients’ symptoms. The authors of these reports suggested that 7-hydroxyamoxapine, amoxapine’s major metabolite, could be implicated in the pathophysiology of these adverse drug reactions due to its dopamine receptor blocking effect.

We did not find any significant reporting association between antidepressant exposure and parkinsonism. However, the association between SRIs and parkinsonism is well documented, although relatively rare [24, 36]. By contrast, the association between TCAs and this movement disorder is more controversial [8]. The association with citalopram was on the border of statistical significance with Bonferroni’s correction (aROR 1.21, 95% CI 1.08–1.36, *p* = 0.0010). This lack of significant association could be explained by the fact that we excluded reports containing more than one antidepressant or by the adjustment on concomitant drugs inducing movement disorders. Further studies are required to precise this mechanism, in particular the involvement of the different 5-HT receptor subtypes [37, 38].

Although bruxism associated with SNRIs has been described [39], it has mainly been documented as a frequent adverse drug reaction of SRIs [40]. Interestingly, in our study, the highest ROR was found with this adverse drug reaction, but the associations were higher with SNRIs (i.e., venlafaxine and duloxetine) than with SRIs (i.e., sertraline, escitalopram, and citalopram). Moreover, the highest level of association was found with vortioxetine, a newly launched antidepressant, with a putative multimodal action as serotonin modulator and stimulator. The pathophysiology of bruxism has been associated to disturbances in the central dopaminergic system [41]. In this context, antidepressant-induced bruxism could be due to an indirect inhibition of dopaminergic pathways due to an increase in extrapyramidal serotonin levels, which could explain why buspirone, a 5–HT1_A_ receptor agonist has shown some efficacy in relieving bruxism [42].

Myoclonus has been mainly reported with TCAs [43, 44], although SRIs have also been associated to this movement disorder [45]. The mechanism of action is imperfectly known but increased serotoninergic transmission could be involved, a study having shown EEG and evoked potentials abnormalities in TCA-induced myoclonus [43]. In our study, it was phenelzine, a MAOI, which was associated with the highest aROR to this movement disorder, followed by clomipramine, a TCA.

The literature concerning antidepressant-induced restless legs syndrome has shown controversial findings [46]. Although some studies have found a link between antidepressants and this movement disorder [46–48], other studies found no association [49] and one study even suggested a potential protective or therapeutic effect of SRIs on restless legs syndrome [50]. As suggested by Fenelon, depression could constitute an important confounding factor when studying the association between antidepressant and restless legs syndrome and analyses should be adjusted on this variable [8]. In our study, mirtazapine was the antidepressant associated with the highest aROR to this movement disorder, an association which has been described [46]. The mechanism of this adverse drug reaction is not precisely known. A SPECT study showed that the severity of restless legs symptoms increased as the availability of the serotonin transporter decreased in the pons and the medulla, highlighting a possible link between an increase serotoninergic neurotransmission in the brainstem and an exacerbation of restless legs syndrome, with a putative dual modulation on striatal dopaminergic neurotransmission and on the activities of spinal motor and sensory neurons [51].

Little is known about the potential association between tics and antidepressants. Some rare case reports have described an association with escitalopram and sertraline [52], fluoxetine [53], paroxetine [54], and with bupropion [55]. We only found an association with tryptophan, an alpha-amino acid which is metabolized into 5-hydroxytryptophan, a precursor of serotonin, which is marketed as an antidepressant in some countries. The pathophysiology of this rare adverse drug reaction is imperfectly known, even though an indirect dopaminergic inhibition through serotoninergic mediation has been proposed [52].

Lastly, antidepressant-induced tremor has been described, in particular with TCAs and SRIs [56, 57], which is in line with our results. We also found an association with fluvoxamine, an SNRI, and with bupropion, an antidepressant derived from amphetamine. The mechanism of this adverse drug reaction is not precisely known but the main putative mechanism of tremorogenic drugs it thought to be an enhancement of the oscillations of peripheral physiological tremor, through an increase in the gain of the muscle receptors and spinal reflex loops, as recalled by Fenelon [8].

### Limitations and strengths of the study

This study presents several limitations. First, despite the important work of the Uppsala Monitoring Center in terms of collection, analyze and checking of reports, the completeness of information collected in VigiBase® is not always guaranteed, and even basic information such as age or sex can be missing. To avoid this bias, we excluded reports in which these data were missing in the adjusted analyses. Other information potentially useful is sometimes missing or incompletely documented, such as patients’ past medical history or certain parameters linked to the drug of interest or to co-medications (i.e., doses, duration of treatment, etc.).

Second, the bias of underreporting is an inherent and systematic limit to this type of pharmacovigilance study [58]. Indeed, the rate of reports can vary according to the type of drug used, the severity of adverse drug reactions, the time of the first occurrence of the adverse drug reaction, the type of notifier, the geographical origin of the report, or the time since the commercial launch of the drug [59]. This also explains why this type of approaches can only provide a very rough and imprecise estimation of adverse drug reactions’ frequencies. However, there is no reason to think that there are some differences in reporting’s rates between “cases” and “non-cases”. Moreover, it has been shown that reporting’s rates were most often similar for drugs belonging to the same pharmacotherapeutic class [60].

Third, we used the MedDRA dictionary for the identification of movement disorders and this tool can lack precision and semiological finesse, especially with complex clinical entities such as movement disorders. Thus, some rare or atypical movement disorders could have been misclassified.

Finally, other limitations should be discussed. This study analyzed data extracted over a 50-year period of time (1967–2017) which means that changes in current medical practices as well as in the understanding and description of the nine selected movement disorders may influence our results. Moreover, an indication bias certainly exists, for instance in patients with Parkinson disease who frequently receive antidepressants during the course of the disease [61, 62]. Information related to medical history of patients is not accessible in VigiBase® and these data could only be approach through proxy, such as co-medications, which we included in our logistic regression models. The case/non-case analysis is an observational and exploratory approach which is useful to detect some safety signals but does not prove causation [60], all the more so we were not able to take into account the level of imputability of the cases in our analyses. Furthermore, while disproportionality was used in this study as a proxy of relative risk, this relation can be discussed [63]. Lastly, it was not possible to include in the analysis pharmacogenetics factors potentially implicated in the occurring of antidepressant-induced movement disorders because this information was missing in VigiBase®. Similarly, we did not study the potential pharmacokinetic interactions between antidepressants and a number of co-medications, in particular antipsychotics, whose blood levels could be increased by inhibition of Cytochrome P450 2D6 (CYP2D6), leading to an increase risk of extrapyramidal symptoms.

This study also presents several strengths. First, VigiBase® is the most important database of pharmacovigilance in the world, with more than 14 million of reports at the time of our study. This allowed to study a rare adverse drug reaction of antidepressants with a unique statistical power as we included more than 600,000 reports in the analysis, this being particularly important in a disproportionality analysis in which the objective is to quantify a signal of risk. Second, the ROR is a reproducible, easy to use and well validated tool to evaluate disproportionality in pharmacovigilance [14, 64]. Third, the ease with which disproportionality studies can be performed appears to be important today, when there is a growing demand for more safe drugs, and they therefore play an important role in the convergence of proofs which allows final decisions in pharmacovigilance [14]. Last, our results are in line with those obtained by Guo et al. through a different approach in a recent nested case-control study focusing on extrapyramidal symptoms [12].

## Conclusion

The present study used the case/non-case approach, a validated method in drug safety research, to precise a relatively rare and little-known adverse effect of antidepressants. The most notified movement disorder after antidepressant exposure was tremor and the highest association was found with bruxism. A potential harmful association was found between movement disorders and use of SRIs in general, and of mirtazapine, vortioxetine, amoxapine, phenelzine, tryptophan, fluvoxamine, citalopram, paroxetine, duloxetine, bupropion, clomipramine, escitalopram, fluoxetine, mianserin, sertraline, venlafaxine and vilazodone in particular.

These results could be useful to help clinicians and patients in making more informed decisions on selecting an appropriate antidepressant. However, this study must be interpreted as an exploratory analysis, and future epidemiological studies should refine these results to precise the frequencies, the clinical impacts and the mechanisms of these adverse drug reactions.

## Supplementary information

**Supplementary file 1.** Complementary tables. **Supplementary Table 1.** List of class of antidepressants adapted from WHO classification. **Supplementary Table 2.** List of movement disorders inducing drugs presented by subtype of movement disorder. **Supplementary Table 3.** List of drugs used to treat the different subtypes of movement disorder. **Supplementary Tables 4.** Results of the case/non-case analyses to rank the signal of the 9 selected movement disorders between different classes of class of antidepressants and between antidepressants. **Supplementary Table 5.** Sensitivity analyses for the antidepressants most frequently reported with the 9 subtypes of movement disorders among 58 antidepressants. For each of these antidepressants, the study period was counted from the year registering its first report in VigiBase® to 1 February, 2017.

## Data Availability

The approval to access the anonymized data maintained by the Uppsala Monitoring Center, located in Sweden requires the data to be treated as confidential with protected and secure access. For this reason, the data cannot be shared publicly. However, if more information about the dataset is requested the authors are happy to provide this.

## Abbreviations

aROR: Adjusted Reporting Odds Ratio
CI: Confidence Interval
MAOI: Monoamine Oxidase Inhibitors
MedDRA: Medical Dictionary for Regulatory Activities
ROR: Reporting Odds Ratio
SNRI: Serotonin-norepinephrine reuptake inhibitors
SRI: Serotonin Reuptake Inhibitors
TCA: Tricyclic Antidepressant
WHO: World Health Organization
