# Supplementary material for: Antidepressants and movement disorders: a postmarketing study in the world pharmacovigilance database
Source: BMC Psychiatry. 2020 Jun 16;20:308. doi: 10.1186/s12888-020-02711-z (PMC7298955; doi:10.1186/s12888-020-02711-z)
Supplement: Supplementary file 1 — Complementary tables. Supplementary Table 1. List of class of antidepressants adapted from WHO classification. Supplementary Table 2. List of movement disorders inducing drugs presented by subtype of movement disorder. Supplementary Table 3. List of drugs used to treat the different subtypes of movement disorder. Supplementary Tables 4. Results of the case/non-case analyses to rank the signal of the 9 selected movement disorders between different classes of class of antidepressants and between antidepressants. Supplementary Table 5. Sensitivity analyses for the antidepressants most frequently reported with the 9 subtypes of movement disorders among 58 antidepressants. For each of these antidepressants, the study period was counted from the year registering its first report in VigiBase® to 1 February, 2017. [file 12888_2020_2711_MOESM1_ESM.docx]

**Supplementary material**

**Supplementary Table 1.** List of class of antidepressants adapted from WHO classification.

| **Tricyclic antidepressant** | **Serotonin reuptake inhibitor** | **Monoamine oxidase inhibitor** | **“Other” antidepressant** |
| --- | --- | --- | --- |
| Amineptine | Citalopram | Iproclozide | Agomelatine |
| Amitriptyline | Escitalopram | Iproniazide | Bifemelane |
| Amoxapine | Etoperidone | Isocarboxazid | Bupropion |
| Butriptyline | Fluoxetine | Moclobemide | Desvenlafaxine |
| Clomipramine | Fluvoxamine | Nialamide | Duloxetine |
| Desipramine | Paroxetine | Phenelzine | Hypericum perforatum |
| Dibenzepin | Sertraline | Toloxatone | Medifoxamine |
| Dosulepin | Zimeldine | Tranylcypromine | Mianserin |
| Doxepin |  |  | Milnacipran |
| Imipramine |  |  | Minaprine |
| Iprindole |  |  | Mirtazapine |
| Lofepramine |  |  | Nefazodone |
| Maprotiline |  |  | Nomifensine |
| Melitracen |  |  | Oxaflozane |
| Nortriptyline |  |  | Oxitriptan |
| Opipramol |  |  | Reboxetine |
| Protriptyline |  |  | Tianeptine |
| Quinupramine |  |  | Trazodone |
| Trimipramine |  |  | Tryptophan |
|  |  |  | Venlafaxine |
|  |  |  | Vilazodone |
|  |  |  | Viloxazine |
|  |  |  | Vortioxetine |

## **Supplementary Table 2.** List of movement disorders inducing drugs presented by subtype of movement disorder.

| **Major category** | **Typical examples of related drug** |
| --- | --- |
| **Akathisia** | |
| Antiepileptics | Carbamazepine, ethosuximide |
| Anxiolytics | Buspirone |
| Cardiovascular drugs | Diltiazem |
| Antipsychotics | Haloperidol, aripiprazole, risperidone |
| Antivertigo preparations | Flunarizine, cinnarizine |
|  | Methysergide |
| Mood stabilizers | Lithium |
| **Bruxism** | |
| Antipsychotics | Haloperidol, chlorpromazine, trifluoperazine |
| Anxiolytics | Hydroxyzine |
| Drugs used in addictive disorders | Nicotine |
| Psychostimulants | Methylphenidate, dexmethylphenidate, atomoxetine, modafinil, armodafinil, theophylline, 8-chlortheophylline, caffeine |
| **Dystonia** | |
| Antiepileptics | Carbamazepine, lamotrigine, phenytoin, fosphenytoin, mephenytoin |
| Gastrointestinal drugs | Metoclopramide |
| Antipsychotics | Haloperidol, thioridazine, levomepromazine, chlorpromazine, sulpiride, levosulpiride, clothiapine, bromperidol |
| Dopa | Levodopa, melevodopa |
| Dopamine agonists | Pramipexole, ropinirole, bromocriptine, pergolide, cabergoline, piribedil, rotigotine, apomorphine, dihydroergocryptine |
| **Myoclonus** | |
| Anesthetics | Chloralose, etomidate, enflurane, propofol |
| Antibiotics/antimalarials | Ceftriaxone, cefotaxime, ceftazidime, ciprofloxacin, levofloxacin, ofloxacin, alatrofloxacin, imipenem, mefloquine, amoxicillin |
| Antiepileptics | Carbamazepine, gabapentin, lamotrigine, phenytoin, fosphenytoin, valproic acid, vigabatrin |
| Antineoplastic drugs | Chlorambucil, ifosfamide |
| Cardiovascular drugs | Diltiazem, nifedipine, verapamil |
| Dopa | Levodopa, melevodopa |
| Dopamine agonists | Pramipexole, ropinirole, bromocriptine, pergolide, cabergoline, piribedil, rotigotine, apomorphine, dihydroergocryptine |
| Gastrointestinal drugs | Bismuth |
| Opioids | Morphine, ethylmorphine, diamorphine, nicomorphine, oxycodone, methadone, levomethadone, normethadone |
| **Parkinsonism** | |
| Antipsychotics | Chlorpromazine, prochlorperazine, perphenazine, fluphenazine, promethazine, isopromethazine, haloperidol, pimozide, sulpiride, levosulpiride, risperidone, olanzapine, ziprasidone |
| Antivertigo preparations | Flunarizine, cinnarizine |
| Antipsychotic and antihypertensive drug | Reserpine |
| **Restless legs syndrome** | |
| Antipsychotics | Aripiprazole, olanzapine, clozapine, quetiapine, risperidone, haloperidol, asenapine, lurasidone |
| Mood stabilizers | Lithium |
| Antiepileptics | Zonisamide, topiramate |
| **Tardive dyskinesia** | |
| Antipsychotics | Haloperidol, perphenazine, thioridazine, chlorpromazine, fluphenazine |
| Anticholinergics | Procyclidine |
| Gastrointestinal drugs | Metoclopramide, prochlorperazine |
| Antiepileptics | Carbamazepine, lamotrigine, phenytoin, fosphenytoin, mephenytoin |
| Decongestants | Phenylpropanolamine |
| Antimalarials | Chloroquine, hydroxychloroquine, amodiaquine |
| Mood stabilizers | Lithium |
| Anxiolytics | Clonazepam, meprobamate, hydroxyzine |
| Drugs used in addictive disorders | Nicotine |
| Psychostimulants | Methylphenidate, dexmethylphenidate, atomoxetine, modafinil, armodafinil, theophylline, 8-chlortheophylline, caffeine |
| **Tics** | |
| Antiepileptics | Carbamazepine, lamotrigine |
| Drugs used in addictive disorders | Amphetamine, dexamphetamine, lisdexamphetamine, hydroxyamphetamine, metamphetamine, dimeamphetamine, tenamphetamine, cocaine, heroin |
| Dopa | Levodopa, melevodopa |
| **Tremor** | |
| Antiarrhythmics | Amiodarone, mexiletine, procainamide |
| Mood stabilizers | Lithium |
| Antiepileptics | Valproic acid |
| Bronchodilators | Salbutamol, levosalbutamol, salmeterol |
| Chemotherapeutics | Tamoxifen, cytarabine, ifosfamide, thalidomide |
| Drugs used in addictive disorders | Cocaine, ethanol, methanol, nicotine |
| Gastrointestinal drugs | Metoclopramide, cimetidine |
| Hormones | Levothyroxine, calcitonin, medroxyprogesterone, epinephrine, norepinephrine |
| Immunosuppressants and immunomodulating agents | Tacrolimus, ciclosporin, interferon alfa, peginterferon alfa |
| Psychostimulants | Theophylline, 8-chlortheophylline, caffeine |
| Antibiotics/antivirals/antifungals | Cytarabine, trimethoprim/sulfamethoxazole, amphotericin B |
| Antipsychotics | Haloperidol, thioridazine, cinnarizine |

## **Supplementary Table 3.** List of drugs used to treat the different subtypes of movement disorder.

| **Major category** | **Related drug** |
| --- | --- |
| **Akathisia** | |
| Anticholinergic agents | Benzatropine, trihexyphenidyl, biperiden |
| Beta blocking agents | Propranolol, metoprolol |
| Antihistamine | Cyproheptadine |
| α_2_ adrenergic agonist | Clonidine |
| Expectorants | Acetylcysteine |
| Anxiolytics | Clonazepam, diazepam, buspirone |
| Psychostimulants | Piracetam |
| Benzoquinolizine | Tetrabenazine |
| Opioids | Naloxone, oxycodone, methadone |
| **Bruxism** | |
| Anxiolytics | Clonazepam |
| Other muscle relaxants, peripherally acting agents | Botulinum toxin (type A and type B) |
| α_2_ adrenergic agonist | Clonidine |
| **Dystonia** | |
| Other muscle relaxants, peripherally acting agents | Botulinum toxin (type A and type B) |
| Dopa | Levodopa, melevodopa |
| Anticholinergic agents | Benzatropine, trihexyphenidyl |
| Benzoquinolizine | Tetrabenazine |
| Muscle relaxants, centrally acting agents | Carisoprodol, chlorzoxazone, cyclobenzaprine, methocarbamol, metaxalone, orphenadrine, baclofen |
| Anxiolytics | Alprazolam, chlordiazepoxide, clonazepam, diazepam |
| **Myoclonus** | |
| Antiepileptics | Primidone, carbamazepine, valproic acid, levetiracetam, phenytoin, ethosuximide |
| Anticholinergic agents | Trihexyphenidyl |
| Anxiolytics | Clonazepam, diazepam |
| Benzoquinolizine | Tetrabenazine |
| Muscle relaxants, centrally acting agents | Baclofen |
| Other muscle relaxants, peripherally acting agents | Botulinum toxin (type A and type B) |
| Antimigraine preparations | Sumatriptan |
| Anesthetics | Oxybate sodium |
| Psychostimulants | Piracetam |
| **Parkinsonism** | |
| Dopa | Levodopa, melevodopa |
| Dopamine agonists | Pramipexole, ropinirole, bromocriptine, pergolide, cabergoline, piribedil, rotigotine, apomorphine, dihydroergocryptine |
| Monoamine oxydase-B inhibitors | Selegiline, rasagiline, safinamide, budipine |
| Catechol O-methyltransferase inhibitors | Entacapone, tolcapone, opicapone, |
| N-methyl-D-aspartate receptor inhibitor | Amantadine |
| Anticholinergic agents | Benzatropine, etybenzatropine, trihexyphenidyl, biperiden, metixene, procyclidine, profenamine, dexetimide, phenglutarimide, mazaticol, bornaprine, tropatepine, orphenadrine |
| **Restless legs syndrome** | |
| Antiepileptics | Gabapentin, pregabalin |
| Dopamine agonists | Pramipexole, ropinirole, rotigotine |
| Opioids | Naloxone, oxycodone, methadone |
| **Tardive dyskinesia** | |
| Benzoquinolizine | Tetrabenazine |
| N-methyl-D-aspartate receptor inhibitor | Amantadine |
| **Tics** | |
| α_2_ adrenergic agonist | Guanfacine |
| Benzoquinolizine | Tetrabenazine |
| Other muscle relaxants, peripherally acting agents | Botulinum toxin (type A and type B) |
| Anxiolytics | Clonazepam |
| Antipsychotics | Fluphenazine, risperidone, aripiprazole |
| Antiepileptics | Topiramate, levetiracetam |
| **Tremor** | |
| Beta blocking agents | Propranolol, atenolol |
| Antiepileptics | Primidone, topiramate, gabapentin |
| Dopa | Levodopa, melevodopa |
| Dopamine agonists | Pramipexole, ropinirole, bromocriptine, pergolide, cabergoline, piribedil, rotigotine, apomorphine, dihydroergocryptine mesylate |
| Anticholinergic agents | Benzatropine, etybenzatropine, trihexyphenidyl, biperiden, metixene, procyclidine, profenamine, dexetimide, phenglutarimide, mazaticol, bornaprine, tropatepine, orphenadrine |
| N-methyl-D-aspartate receptor inhibitor | Amantadine |
| Antipsychotics | Clozapine |

**References for Supplementary Tables 2 and 3.**

Allen RP. Restless legs syndrome/Willis Ekbom disease: evaluation and treatment. Int Rev Psychiatry. 2014 Apr;26(2):248–62.

Cornett EM, Novitch M, Kaye AD, Kata V, Kaye AM. Medication-Induced Tardive Dyskinesia: A Review and Update. Ochsner J. 2017;17(2):162–74.

Elias WJ, Shah BB. Tremor. JAMA. 2014 Mar 5;311(9):948–54.

Falisi G, Rastelli C, Panti F, Maglione H, Quezada Arcega R. Psychotropic drugs and bruxism. Expert Opin Drug Saf. 2014 Oct;13(10):1319–26.

Gazewood JD, Richards DR, Clebak K. Parkinson disease: an update. Am Fam Physician. 2013 Feb 15;87(4):267–73.

Guaita M, Högl B. Current Treatments of Bruxism. Curr Treat Options Neurol. 2016 Feb;18(2):10.

Jankovic J. Therapeutic Developments for Tics and Myoclonus. Mov Disord. 2015 Sep 15;30(11):1566–73.

Jinnah HA, Factor SA. Diagnosis and treatment of dystonia. Neurol Clin. 2015 Feb;33(1):77–100.

Lohr JB, Eidt CA, Abdulrazzaq Alfaraj A, Soliman MA. The clinical challenges of akathisia. CNS Spectr. 2015 Dec;20 Suppl 1:1–14; quiz 15–6.

Mehta SH, Morgan JC, Sethi KD. Drug-induced movement disorders. Neurol Clin. 2015 Feb;33(1):153–74.

Morgan JC, Sethi KD. Drug-induced tremors. Lancet Neurol. 2005 Dec;4(12):866–76.

Patatanian E, Claborn MK. Drug-Induced Restless Legs Syndrome. Ann Pharmacother. 2018 Jul;52(7):662–72.

Seigneurie A-S, Sauvanaud F, Limosin F. [Prevention and treatment of tardive dyskinesia caused by antipsychotic drugs]. Encephale. 2016 Jun;42(3):248–54.

Shin H-W, Chung SJ. Drug-induced parkinsonism. J Clin Neurol. 2012 Mar;8(1):15–21.

Spina E, Sturiale V, Valvo S, Ancione M, Di Rosa AE, Meduri M, et al. Prevalence of acute dystonic reactions associated with neuroleptic treatment with and without anticholinergic prophylaxis. Int Clin Psychopharmacol. 1993;8(1):21–4.

Tarsy D, Simon DK. Dystonia. N Engl J Med. 2006 Aug 24;355(8):818–29.

Woods SW, Morgenstern H, Saksa JR, Walsh BC, Sullivan MC, Money R, et al. Incidence of tardive dyskinesia with atypical versus conventional antipsychotic medications: a prospective cohort study. J Clin Psychiatry. 2010 Apr;71(4):463–74.

**Supplementary Tables 4.** Results of the case/non-case analyses to rank the signal of the 9 selected movement disorders between different classes of class of antidepressants and between antidepressants.

**Supplementary Table 4.a.** Akathisia.

| Antidepressants | Number of reports  (n = 625,167) | Case  (n = 1250) | Non-case  (n = 623,917) | Adjusted ROR*  [95% CI] | *p-value* |
| --- | --- | --- | --- | --- | --- |
| Tricyclic antidepressants | 103,139 | 87 | 103,052 | 0.40 [0.32 – 0.49] | <.0001 |
| Serotonin reuptake inhibitors* | 291,020 | 730 | 290,290 | 1.50 [1.34 – 1.68] | <.0001 |
| Monoamine oxidase inhibitors | 7447 | 8 | 7439 | 0.51 [0.25 – 1.02] | 0.0581 |
| “Other” antidepressants | 223,561 | 425 | 223,136 | 0.98 [0.88 – 1.11] | 0.7921 |
| Agomelatine | 1653 | 4 | 1649 | 1.31 [0.49 – 3.50] | 0.5934 |
| Amineptine | 926 | 0 | 926 |  |  |
| Amitriptyline | 49,707 | 21 | 49,686 | 0.22 [0.14 – 0.34] | <.0001 |
| Amoxapine | 1269 | 8 | 1261 | 3.22 [1.60 – 6.48] | 0.0010 |
| Bifemelane | 19 | 0 | 19 |  |  |
| Bupropion | 46,160 | 33 | 46,127 | 0.32 [0.23 – 0.46] | <.0001 |
| Butriptyline | 12 | 0 | 12 |  |  |
| Citalopram* | 85,364 | 249 | 85,115 | 1.60 [1.39 – 1.84] | <.0001 |
| Clomipramine | 8356 | 13 | 8343 | 0.63 [0.36 – 1.08] | 0.0937 |
| Desipramine | 2405 | 0 | 2405 |  |  |
| Desvenlafaxine | 5976 | 7 | 5969 | 0.62 [0.30 – 1.31] | 0.2143 |
| Dibenzepin | 229 | 0 | 229 |  |  |
| Dosulepin | 4885 | 4 | 4881 | 0.47 [0.18 – 1.26] | 0.1334 |
| Doxepin | 7838 | 7 | 7831 | 0.47 [0.22 – 0.99] | 0.0457 |
| Duloxetine | 43,293 | 85 | 43,208 | 1.15 [0.92 – 1.43] | 0.2207 |
| Escitalopram* | 34,693 | 136 | 34,557 | 2.02 [1.69 – 2.42] | <.0001 |
| Etoperidone | 19 | 0 | 19 |  |  |
| Fluoxetine | 70,325 | 158 | 70,167 | 1.02 [0.86 – 1.20] | 0.8432 |
| Fluvoxamine | 8062 | 32 | 8030 | 1.61 [1.13 – 2.29] | 0.0086 |
| Hypericum perforatum | 2238 | 0 | 2238 |  |  |
| Imipramine | 9972 | 13 | 9959 | 0.58 [0.34 – 1.01] | 0.0528 |
| Iprindole | 82 | 0 | 82 |  |  |
| Iproclozide | 0 |  |  |  |  |
| Iproniazide | 105 | 0 | 105 |  |  |
| Isocarboxazid | 162 | 0 | 162 |  |  |
| Lofepramine | 3392 | 4 | 3388 | 0.63 [0.24 – 1.68] | 0.3561 |
| Maprotiline | 3410 | 8 | 3402 | 1.23 [0.61 – 2.46] | 0.5656 |
| Medifoxamine | 65 | 0 | 65 |  |  |
| Melitracen | 681 | 1 | 680 | 0.95 [0.13 – 6.80] | 0.9629 |
| Mianserin* | 6049 | 24 | 6025 | 2.49 [1.66 – 3.74] | <.0001 |
| Milnacipran | 2595 | 3 | 2592 | 0.71 [0.23 – 2.22] | 0.5605 |
| Minaprine | 94 | 0 | 94 |  |  |
| Mirtazapine* | 20,941 | 96 | 20,845 | 2.55 [2.06 – 3.14] | <.0001 |
| Moclobemide | 3176 | 5 | 3171 | 0.83 [0.35 – 2.00] | 0.6791 |
| Nefazodone | 5523 | 10 | 5513 | 0.88 [0.47 – 1.65] | 0.6940 |
| Nialamide | 26 | 0 | 26 |  |  |
| Nomifensine | 1311 | 0 | 1311 |  |  |
| Nortriptyline | 8484 | 8 | 8476 | 0.48 [0.24 – 0.97] | 0.0410 |
| Opipramol | 992 | 0 | 992 |  |  |
| Oxaflozane | 2 | 0 | 2 |  |  |
| Oxitriptan | 178 | 0 | 178 |  |  |
| Paroxetine* | 58,491 | 166 | 58,325 | 1.49 [1.27 – 1.76] | <.0001 |
| Phenelzine | 2364 | 3 | 2361 | 0.57 [0.18 – 1.76] | 0.3259 |
| Protriptyline | 479 | 0 | 479 |  |  |
| Quinupramine | 20 | 0 | 20 |  |  |
| Reboxetine | 1447 | 6 | 1441 | 1.95 [0.87 – 4.36] | 0.1044 |
| Sertraline | 67,840 | 125 | 67,715 | 0.85 [0.71 – 1.02] | 0.0854 |
| Tianeptine | 1888 | 1 | 1887 | 0.35 [0.05 – 2.45] | 0.2874 |
| Toloxatone | 87 | 0 | 87 |  |  |
| Tranylcypromine | 1527 | 0 | 1527 |  |  |
| Trazodone | 19,403 | 44 | 19,359 | 1.08 [0.80 – 1.46] | 0.6249 |
| Trimipramine | 2424 | 5 | 2419 | 1.06 [0.44 – 2.55] | 0.9010 |
| Tryptophan | 12,726 | 2 | 12,724 | 0.10 [0.03 – 0.41] | 0.0013 |
| Venlafaxine | 53,563 | 107 | 53,456 | 0.98 [0.80 – 1.19] | 0.8234 |
| Vilazodone | 2111 | 3 | 2108 | 0.75 [0.24 – 2.32] | 0.6122 |
| Viloxazine | 525 | 0 | 525 |  |  |
| Vortioxetine | 1787 | 7 | 1780 | 2.17 [1.03 – 4.57] | 0.0418 |
| Zimeldine | 919 | 0 | 919 |  |  |

*CI confidence interval, ROR reporting odd ratio.*

^a^Adjusted ROR were calculated in adjusted univariate logistic regression analysis, with adjustment for age, gender, drugs used in akathisia and drugs associated with akathisia.

* Significant signal was defined as adjusted ROR > 1 with α threshold of 0.001, and the number of cases being at least 10.

**Supplementary Table 4.b.** Bruxism.

| Antidepressants | Number of reports  (n = 625,167) | Case  (n = 722) | Non-case  (n = 624,445) | Adjusted ROR*  [95% CI] | *p-value* |
| --- | --- | --- | --- | --- | --- |
| Tricyclic antidepressants | 103,139 | 17 | 103,122 | 0.13 [0.08 – 0.21] | <.0001 |
| Serotonin reuptake inhibitors* | 291,020 | 427 | 290,593 | 1.56 [1.35 – 1.82] | <.0001 |
| Monoamine oxidase inhibitors | 7447 | 3 | 7444 | 0.34 [0.11 – 1.07] | 0.0655 |
| “Other” antidepressants | 223,561 | 275 | 223,286 | 1.14 [0.98 – 1.33] | 0.0808 |
| Agomelatine | 1653 | 2 | 1651 | 1.02 [0.26 – 4.11] | 0.9728 |
| Amineptine | 926 | 0 | 926 |  |  |
| Amitriptyline | 49,707 | 8 | 49,699 | 0.14 [0.07 – 0.29] | <.0001 |
| Amoxapine | 1269 | 0 | 1269 |  |  |
| Bifemelane | 19 | 0 | 19 |  |  |
| Bupropion | 46,160 | 40 | 46,120 | 0.67 [0.48 – 0.92] | 0.0122 |
| Butriptyline | 12 | 0 | 12 |  |  |
| Citalopram* | 85,364 | 133 | 85,231 | 1.48 [1.22 – 1.78] | <.0001 |
| Clomipramine | 8356 | 2 | 8354 | 0.19 [0.05 – 0.77] | 0.0200 |
| Desipramine | 2405 | 0 | 2405 |  |  |
| Desvenlafaxine | 5976 | 14 | 5962 | 2.01 [1.18 – 3.41] | 0.0101 |
| Dibenzepin | 229 | 0 | 229 |  |  |
| Dosulepin | 4885 | 2 | 4883 | 0.40 [0.10 – 1.59] | 0.1907 |
| Doxepin | 7838 | 1 | 7837 | 0.12 [0.02 – 0.88] | 0.0365 |
| Duloxetine* | 43,293 | 82 | 43,211 | 1.83 [1.46 – 2.31] | <.0001 |
| Escitalopram* | 34,693 | 63 | 34,630 | 1.62 [1.25 – 2.10] | 0.0003 |
| Etoperidone | 19 | 0 | 19 |  |  |
| Fluoxetine | 70,325 | 62 | 70,263 | 0.66 [0.51 – 0.85] | 0.0015 |
| Fluvoxamine | 8062 | 13 | 8049 | 1.28 [0.74 – 2.22] | 0.3791 |
| Hypericum perforatum | 2238 | 0 | 2238 |  |  |
| Imipramine | 9972 | 1 | 9971 | 0.08 [0.01 – 0.58] | 0.0121 |
| Iprindole | 82 | 0 | 82 |  |  |
| Iproclozide | 0 |  |  |  |  |
| Iproniazide | 105 | 0 | 105 |  |  |
| Isocarboxazid | 162 | 0 | 162 |  |  |
| Lofepramine | 3392 | 0 | 3392 |  |  |
| Maprotiline | 3410 | 0 | 3410 |  |  |
| Medifoxamine | 65 | 0 | 65 |  |  |
| Melitracen | 681 | 0 | 681 |  |  |
| Mianserin | 6049 | 0 | 6049 |  |  |
| Milnacipran | 2595 | 1 | 2594 | 0.35 [0.05 – 2.50] | 0.2967 |
| Minaprine | 94 | 0 | 94 |  |  |
| Mirtazapine | 20,941 | 9 | 20,932 | 0.42 [0.22 – 0.82] | 0.0107 |
| Moclobemide | 3176 | 1 | 3175 | 0.29 [0.04 – 2.04] | 0.2116 |
| Nefazodone | 5523 | 0 | 5523 |  |  |
| Nialamide | 26 | 0 | 26 |  |  |
| Nomifensine | 1311 | 0 | 1311 |  |  |
| Nortriptyline | 8484 | 3 | 8481 | 0.33 [0.11 – 1.01] | 0.0524 |
| Opipramol | 992 | 0 | 992 |  |  |
| Oxaflozane | 2 | 0 | 2 |  |  |
| Oxitriptan | 178 | 0 | 178 |  |  |
| Paroxetine | 58,491 | 95 | 58,396 | 1.43 [1.15 – 1.78] | 0.0011 |
| Phenelzine | 2364 | 0 | 2364 |  |  |
| Protriptyline | 479 | 0 | 479 |  |  |
| Quinupramine | 20 | 0 | 20 |  |  |
| Reboxetine | 1447 | 1 | 1446 | 0.58 [0.08 – 4.09] | 0.5806 |
| Sertraline* | 67,840 | 124 | 67,716 | 1.63 [1.35 – 1.98] | <.0001 |
| Tianeptine | 1888 | 0 | 1888 |  |  |
| Toloxatone | 87 | 0 | 87 |  |  |
| Tranylcypromine | 1527 | 2 | 1525 | 1.11 [0.28 – 4.47] | 0.8798 |
| Trazodone | 19,403 | 9 | 19,394 | 0.44 [0.23 – 0.85] | 0.0146 |
| Trimipramine | 2424 | 1 | 2423 | 0.40 [0.06 – 2.81] | 0.3550 |
| Tryptophan | 12,726 | 1 | 12,725 | 0.08 [0.01 – 0.54] | 0.0101 |
| Venlafaxine* | 53,563 | 115 | 53,448 | 1.98 [1.63 – 2.42] | <.0001 |
| Vilazodone | 2111 | 5 | 2106 | 2.04 [0.85 – 4.92] | 0.1121 |
| Viloxazine | 525 | 0 | 525 |  |  |
| Vortioxetine* | 1787 | 10 | 1777 | 4.71 [2.52 – 8.80] | <.0001 |
| Zimeldine | 919 | 0 | 919 |  |  |

*CI confidence interval, ROR reporting odd ratio.*

^a^Adjusted ROR were calculated in adjusted univariate logistic regression analysis, with adjustment for age, gender, drugs used in bruxism and drugs associated with bruxism.

* Significant signal was defined as adjusted ROR > 1 with α threshold of 0.001, and the number of case being at least 10.

**Supplementary Table 4.c.** Dystonia.

| Antidepressants | Number of reports  (n = 625,167) | Case  (n = 3428) | Non-case  (n = 621,739) | Adjusted ROR*  [95% CI] | *p-value* |
| --- | --- | --- | --- | --- | --- |
| Tricyclic antidepressants | 103,139 | 490 | 102,649 | 0.88 [0.80 – 0.97] | 0.0125 |
| Serotonin reuptake inhibitors* | 291,020 | 2096 | 288,924 | 1.66 [1.55 – 1.78] | <.0001 |
| Monoamine oxidase inhibitors | 7447 | 39 | 7408 | 0.96 [0.70 – 1.32] | 0.7971 |
| “Other” antidepressants | 223,561 | 803 | 222,758 | 0.59 [0.54 – 0.64] | <.0001 |
| Agomelatine | 1653 | 3 | 1650 | 0.34 [0.11 – 1.05] | 0.0611 |
| Amineptine | 926 | 2 | 924 | 0.41 [0.10 – 1.62] | 0.2019 |
| Amitriptyline | 49,707 | 163 | 49,544 | 0.65 [0.56 – 0.76] | <.0001 |
| Amoxapine* | 1269 | 30 | 1239 | 4.43 [3.07 – 6.39] | <.0001 |
| Bifemelane | 19 | 0 | 19 |  |  |
| Bupropion | 46,160 | 112 | 46,048 | 0.38 [0.32 – 0.46] | <.0001 |
| Butriptyline | 12 | 0 | 12 |  |  |
| Citalopram | 85,364 | 434 | 84,930 | 0.95 [0.85 – 1.05] | 0.2742 |
| Clomipramine | 8356 | 72 | 8284 | 1.33 [1.05 – 1.69] | 0.0165 |
| Desipramine | 2405 | 15 | 2390 | 0.87 [0.52 – 1.44] | 0.5832 |
| Desvenlafaxine | 5976 | 21 | 5955 | 0.66 [0.43 – 1.02] | 0.0584 |
| Dibenzepin | 229 | 0 | 229 |  |  |
| Dosulepin | 4885 | 27 | 4858 | 1.18 [0.80 – 1.72] | 0.4021 |
| Doxepin | 7838 | 30 | 7808 | 0.79 [0.55 – 1.14] | 0.2097 |
| Duloxetine | 43,293 | 124 | 43,169 | 0.58 [0.48 – 0.69] | <.0001 |
| Escitalopram | 34,693 | 160 | 34,533 | 0.81 [0.69 – 0.95] | 0.0083 |
| Etoperidone | 19 | 0 | 19 |  |  |
| Fluoxetine* | 70,325 | 621 | 69,704 | 1.51 [1.39 – 1.65] | <.0001 |
| Fluvoxamine* | 8062 | 79 | 7983 | 1.48 [1.18 – 1.86] | 0.0006 |
| Hypericum perforatum | 2238 | 3 | 2235 | 0.26 [0.09 – 0.82] | 0.0208 |
| Imipramine | 9972 | 75 | 9897 | 1.17 [0.93 – 1.48] | 0.1753 |
| Iprindole | 82 | 1 | 81 | 2.01 [0.28 – 14.52] | 0.4873 |
| Iproclozide | 0 |  |  |  |  |
| Iproniazide | 105 | 0 | 105 |  |  |
| Isocarboxazid | 162 | 0 | 162 |  |  |
| Lofepramine | 3392 | 11 | 3381 | 0.61 [0.34 – 1.11] | 0.1055 |
| Maprotiline | 3410 | 9 | 3401 | 0.49 [0.25 – 0.93] | 0.0305 |
| Medifoxamine | 65 | 0 | 65 |  |  |
| Melitracen | 681 | 6 | 675 | 2.14 [0.95 – 4.79] | 0.0653 |
| Mianserin | 6049 | 16 | 6033 | 0.64 [0.39 – 1.05] | 0.0746 |
| Milnacipran | 2595 | 4 | 2591 | 0.33 [0.13 – 0.89] | 0.0282 |
| Minaprine | 94 | 1 | 93 | 2.12 [0.29 – 15.30] | 0.4563 |
| Mirtazapine | 20,941 | 93 | 20,848 | 0.94 [0.76 – 1.15] | 0.5267 |
| Moclobemide | 3176 | 18 | 3158 | 1.13 [0.71 – 1.80] | 0.6145 |
| Nefazodone | 5523 | 22 | 5501 | 0.73 [0.48 – 1.11] | 0.1440 |
| Nialamide | 26 | 0 | 26 |  |  |
| Nomifensine | 1311 | 4 | 1307 | 0.58 [0.22 – 1.56] | 0.2814 |
| Nortriptyline | 8484 | 42 | 8442 | 0.98 [0.72 – 1.33] | 0.8826 |
| Opipramol | 992 | 3 | 989 | 0.66 [0.21 – 2.04] | 0.4650 |
| Oxaflozane | 2 | 0 | 2 |  |  |
| Oxitriptan | 178 | 1 | 177 | 0.86 [0.12 – 6.16] | 0.8781 |
| Paroxetine* | 58,491 | 558 | 57,933 | 1.87 [1.70 – 2.05] | <.0001 |
| Phenelzine | 2364 | 12 | 2352 | 0.85 [0.48 – 1.50] | 0.5731 |
| Protriptyline | 479 | 4 | 475 | 1.45 [0.54 – 3.90] | 0.4573 |
| Quinupramine | 20 | 0 | 20 |  |  |
| Reboxetine | 1447 | 6 | 1441 | 0.70 [0.31 – 1.55] | 0.3756 |
| Sertraline | 67,840 | 402 | 67,438 | 1.02 [0.92 – 1.13] | 0.7058 |
| Tianeptine | 1888 | 2 | 1886 | 0.28 [0.07 – 1.12] | 0.0716 |
| Toloxatone | 87 | 0 | 87 |  |  |
| Tranylcypromine | 1527 | 9 | 1518 | 1.06 [0.55 – 2.04] | 0.8675 |
| Trazodone | 19,403 | 100 | 19,303 | 1.01 [0.83 – 1.24] | 0.9157 |
| Trimipramine | 2424 | 19 | 2405 | 1.57 [1.00 – 2.47] | 0.0513 |
| Tryptophan | 12,726 | 4 | 12,722 | 0.07 [0.03 – 0.19] | <.0001 |
| Venlafaxine | 53,563 | 301 | 53,262 | 1.02 [0.90 – 1.15] | 0.7847 |
| Vilazodone | 2111 | 2 | 2109 | 0.18 [0.05 – 0.72] | 0.0150 |
| Viloxazine | 525 | 0 | 525 |  |  |
| Vortioxetine | 1787 | 5 | 1782 | 0.51 [0.21 – 1.23] | 0.1331 |
| Zimeldine | 919 | 2 | 917 | 0.47 [0.12 – 1.90] | 0.2920 |

*CI confidence interval, ROR reporting odd ratio.*

^a^Adjusted ROR were calculated in adjusted univariate logistic regression analysis, with adjustment for age, gender, drugs used in dystonia and drugs associated with dystonia.

* Significant signal was defined as adjusted ROR > 1 with α threshold of 0.001, and the number of case being at least 10.

**Supplementary Table 4.d.** Myoclonus.

| Antidepressants | Number of reports  (n = 625,167) | Case  (n = 1229) | Non-case  (n = 623,938) | Adjusted ROR*  [95% CI] | *p-value* |
| --- | --- | --- | --- | --- | --- |
| Tricyclic antidepressants | 103,139 | 183 | 102,956 | 0.87 [0.74 – 1.02] | 0.0803 |
| Serotonin reuptake inhibitors* | 291,020 | 638 | 290,382 | 1.25 [1.12 – 1.40] | <.0001 |
| Monoamine oxidase inhibitors | 7447 | 35 | 7412 | 2.54 [1.81 – 3.56] | <.0001 |
| “Other” antidepressants | 223,561 | 373 | 223,188 | 0.78 [0.69 – 0.88] | <.0001 |
| Agomelatine | 1653 | 4 | 1649 | 1.30 [0.49 – 3.47] | 0.6038 |
| Amineptine | 926 | 2 | 924 | 1.16 [0.29 – 4.64] | 0.8376 |
| Amitriptyline | 49,707 | 90 | 49,617 | 0.88 [0.71 – 1.09] | 0.2305 |
| Amoxapine | 1269 | 3 | 1266 | 1.26 [0.41 – 3.93] | 0.6874 |
| Bifemelane | 19 | 0 | 19 |  |  |
| Bupropion | 46,160 | 43 | 46,117 | 0.46 [0.34 – 0.63] | <.0001 |
| Butriptyline | 12 | 0 | 12 |  |  |
| Citalopram* | 85,364 | 215 | 85,149 | 1.31 [1.13 – 1.52] | 0.0003 |
| Clomipramine* | 8356 | 43 | 8313 | 2.73 [2.01 – 3.71] | <.0001 |
| Desipramine | 2405 | 4 | 2401 | 0.86 [0.32 – 2.29] | 0.7569 |
| Desvenlafaxine | 5976 | 2 | 5974 | 0.18 [0.05 – 0.72] | 0.0149 |
| Dibenzepin | 229 | 0 | 229 |  |  |
| Dosulepin | 4885 | 3 | 4882 | 0.32 [0.10 – 0.98] | 0.0464 |
| Doxepin | 7838 | 6 | 7832 | 0.37 [0.17 – 0.84] | 0.0164 |
| Duloxetine | 43,293 | 64 | 43,229 | 0.74 [0.58 – 0.96] | 0.0210 |
| Escitalopram | 34,693 | 63 | 34,630 | 0.91 [0.71 – 1.17] | 0.4697 |
| Etoperidone | 19 | 0 | 19 |  |  |
| Fluoxetine | 70,325 | 136 | 70,189 | 1.02 [0.85 – 1.22] | 0.8649 |
| Fluvoxamine | 8062 | 28 | 8034 | 1.81 [1.25 – 2.64] | 0.0019 |
| Hypericum perforatum | 2238 | 3 | 2235 | 0.72 [0.23 – 2.24] | 0.5725 |
| Imipramine | 9972 | 15 | 9957 | 0.77 [0.46 – 1.28] | 0.3147 |
| Iprindole | 82 | 0 | 82 |  |  |
| Iproclozide | 0 |  |  |  |  |
| Iproniazide | 105 | 1 | 104 | 4.92 [0.69 – 35.06] | 0.1115 |
| Isocarboxazid | 162 | 1 | 161 | 3.31 [0.46 – 23.59] | 0.2333 |
| Lofepramine | 3392 | 2 | 3390 | 0.31 [0.08 – 1.25] | 0.1001 |
| Maprotiline | 3410 | 6 | 3404 | 0.96 [0.43 – 2.14] | 0.9146 |
| Medifoxamine | 65 | 0 | 65 |  |  |
| Melitracen | 681 | 0 | 681 |  |  |
| Mianserin | 6049 | 18 | 6031 | 1.52 [0.96 – 2.43] | 0.0771 |
| Milnacipran | 2595 | 2 | 2593 | 0.41 [0.10 – 1.63] | 0.2030 |
| Minaprine | 94 | 0 | 94 |  |  |
| Mirtazapine* | 20,941 | 69 | 20,872 | 1.61 [1.26 – 2.06] | 0.0001 |
| Moclobemide | 3176 | 5 | 3171 | 0.83 [0.34 – 1.99] | 0.6721 |
| Nefazodone | 5523 | 3 | 5520 | 0.28 [0.09 – 0.88] | 0.0288 |
| Nialamide | 26 | 0 | 26 |  |  |
| Nomifensine | 1311 | 0 | 1311 |  |  |
| Nortriptyline | 8484 | 6 | 8478 | 0.34 [0.15 – 0.77] | 0.0090 |
| Opipramol | 992 | 1 | 991 | 0.53 [0.07 – 3.73] | 0.5192 |
| Oxaflozane | 2 | 0 | 2 |  |  |
| Oxitriptan | 178 | 1 | 177 | 3.02 [0.42 – 21.71] | 0.2714 |
| Paroxetine* | 58,491 | 155 | 58,336 | 1.43 [1.21 – 1.69] | <.0001 |
| Phenelzine* | 2364 | 21 | 2343 | 4.85 [3.14 – 7.49] | <.0001 |
| Protriptyline | 479 | 1 | 478 | 1.15 [0.16 – 8.20] | 0.8883 |
| Quinupramine | 20 | 1 | 19 | 26.74 [3.57 – 200.29] | 0.0014 |
| Reboxetine | 1447 | 1 | 1446 | 0.36 [0.05 – 2.52] | 0.3006 |
| Sertraline | 67,840 | 102 | 67,738 | 0.74 [0.61 – 0.91] | 0.0037 |
| Tianeptine | 1888 | 6 | 1882 | 1.58 [0.71 – 3.53] | 0.2668 |
| Toloxatone | 87 | 1 | 86 | 5.86 [0.82 – 42.07] | 0.0789 |
| Tranylcypromine | 1527 | 6 | 1521 | 2.11 [0.94 – 4.71] | 0.0691 |
| Trazodone | 19,403 | 20 | 19,383 | 0.48 [0.31 – 0.74] | 0.0010 |
| Trimipramine | 2424 | 3 | 2421 | 0.63 [0.20 – 1.95] | 0.4178 |
| Tryptophan | 12,726 | 1 | 12,725 | 0.05 [0.01 – 0.28] | 0.0012 |
| Venlafaxine | 53,563 | 132 | 53,431 | 1.31 [1.09 – 1.57] | 0.0036 |
| Vilazodone | 2111 | 0 | 2111 |  |  |
| Viloxazine | 525 | 5 | 520 | 4.66 [1.93 – 11.28] | 0.0006 |
| Vortioxetine | 1787 | 1 | 1786 | 0.31 [0.04 – 2.15] | 0.2337 |
| Zimeldine | 919 | 2 | 917 | 1.22 [0.30 – 4.87] | 0.7836 |

*CI confidence interval, ROR reporting odd ratio.*

^a^Adjusted ROR were calculated in adjusted univariate logistic regression analysis, with adjustment for age, gender, drugs used in myoclonus and drugs associated with myoclonus.

* Significant signal was defined as adjusted ROR > 1 with α threshold of 0.001, and the number of case being at least 10.

**Supplementary Table 4.e.** Parkinsonism.

| Antidepressants | Number of reports  (n = 625,167) | Case  (n = 2077) | Non-case  (n = 623,090) | Adjusted ROR*  [95% CI] | *p-value* |
| --- | --- | --- | --- | --- | --- |
| Tricyclic antidepressants | 103,139 | 298 | 102,841 | 0.77 [0.68 – 0.87] | <.0001 |
| Serotonin reuptake inhibitors* | 291,020 | 1075 | 289,945 | 1.24 [1.14 – 1.35] | <.0001 |
| Monoamine oxidase inhibitors | 7447 | 25 | 7422 | 1.12 [0.75 – 1.66] | 0.5874 |
| “Other” antidepressants | 223,561 | 679 | 222,882 | 0.92 [0.84 – 1.01] | 0.0670 |
| Agomelatine | 1653 | 5 | 1648 | 1.06 [0.44 – 2.57] | 0.8905 |
| Amineptine | 926 | 3 | 923 | 1.03 [0.33 – 3.22] | 0.9555 |
| Amitriptyline | 49,707 | 124 | 49,583 | 0.67 [0.56 – 0.80] | <.0001 |
| Amoxapine | 1269 | 9 | 1260 | 2.02 [1.04 – 3.92] | 0.0370 |
| Bifemelane | 19 | 1 | 18 | 8.28 [1.06 – 64.44] | 0.0435 |
| Bupropion | 46,160 | 90 | 46,070 | 0.75 [0.61 – 0.93] | 0.0084 |
| Butriptyline | 12 | 0 | 12 |  |  |
| Citalopram | 85,364 | 361 | 85,003 | 1.21 [1.08 – 1.36] | 0.0010 |
| Clomipramine | 8356 | 40 | 8316 | 1.33 [0.97 – 1.82] | 0.0763 |
| Desipramine | 2405 | 5 | 2400 | 0.62 [0.26 – 1.50] | 0.2885 |
| Desvenlafaxine | 5976 | 10 | 5966 | 0.64 [0.35 – 1.20] | 0.1643 |
| Dibenzepin | 229 | 0 | 229 |  |  |
| Dosulepin | 4885 | 11 | 4874 | 0.61 [0.34 – 1.11] | 0.1065 |
| Doxepin | 7838 | 24 | 7814 | 0.82 [0.82 – 1.22] | 0.3218 |
| Duloxetine | 43,293 | 112 | 43,181 | 0.84 [0.70 – 1.02] | 0.0812 |
| Escitalopram | 34,693 | 138 | 34,555 | 1.16 [0.97 – 1.38] | 0.0992 |
| Etoperidone | 19 | 0 | 19 |  |  |
| Fluoxetine | 70,325 | 198 | 70,127 | 0.94 [0.81 – 1.09] | 0.3850 |
| Fluvoxamine | 8062 | 30 | 8032 | 1.05 [0.73 – 1.52] | 0.7778 |
| Hypericum perforatum | 2238 | 4 | 2234 | 0.70 [0.26 – 1.86] | 0.4678 |
| Imipramine | 9972 | 23 | 9949 | 0.65 [0.43 – 0.97] | 0.0370 |
| Iprindole | 82 | 0 | 82 |  |  |
| Iproclozide | 0 |  |  |  |  |
| Iproniazide | 105 | 2 | 103 | 4.61 [1.10 – 19.37] | 0.0367 |
| Isocarboxazid | 162 | 0 | 162 |  |  |
| Lofepramine | 3392 | 9 | 3383 | 0.72 [0.37 – 1.39] | 0.3288 |
| Maprotiline | 3410 | 19 | 3391 | 1.66 [1.05 – 2.61] | 0.0303 |
| Medifoxamine | 65 | 0 | 65 |  |  |
| Melitracen | 681 | 9 | 672 | 3.87 [2.00 – 7.50] | <.0001 |
| Mianserin | 6049 | 20 | 6029 | 0.75 [0.48 – 1.16] | 0.1938 |
| Milnacipran | 2595 | 5 | 2590 | 0.67 [0.28 – 1.60] | 0.3646 |
| Minaprine | 94 | 1 | 93 | 2.77 [0.38 – 20.05] | 0.3132 |
| Mirtazapine | 20,941 | 113 | 20,828 | 1.24 [1.02 – 1.50] | 0.0296 |
| Moclobemide | 3176 | 11 | 3165 | 1.09 [0.60 – 1.97] | 0.7807 |
| Nefazodone | 5523 | 16 | 5507 | 1.09 [0.66 – 1.78] | 0.7414 |
| Nialamide | 26 | 0 | 26 |  |  |
| Nomifensine | 1311 | 0 | 1311 |  |  |
| Nortriptyline | 8484 | 20 | 8464 | 0.63 [0.41 – 0.98] | 0.0412 |
| Opipramol | 992 | 2 | 990 | 0.61 [0.15 – 2.43] | 0.4799 |
| Oxaflozane | 2 | 0 | 2 |  |  |
| Oxitriptan | 178 | 2 | 176 | 3.68 [0.91 – 14.97] | 0.0684 |
| Paroxetine | 58,491 | 214 | 58,277 | 1.16 [1.01 – 1.34] | 0.0424 |
| Phenelzine | 2364 | 8 | 2356 | 1.28 [0.64 – 2.58] | 0.4834 |
| Protriptyline | 479 | 0 | 479 |  |  |
| Quinupramine | 20 | 0 | 20 |  |  |
| Reboxetine | 1447 | 3 | 1444 | 0.69 [0.22 – 2.14] | 0.5156 |
| Sertraline | 67,840 | 272 | 67,568 | 1.23 [1.08 – 1.39] | 0.0019 |
| Tianeptine | 1888 | 6 | 1882 | 0.75 [0.34 – 1.68] | 0.4869 |
| Toloxatone | 87 | 0 | 87 |  |  |
| Tranylcypromine | 1527 | 4 | 1523 | 0.87 [0.32 – 2.32] | 0.7776 |
| Trazodone | 19,403 | 86 | 19,317 | 1.03 [0.83 – 1.28] | 0.7803 |
| Trimipramine | 2424 | 5 | 2419 | 0.52 [0.22 – 1.25] | 0.1448 |
| Tryptophan | 12,726 | 2 | 12,724 | 0.05 [0.01 – 0.20] | <.0001 |
| Venlafaxine | 53,563 | 199 | 53,364 | 1.22 [1.06 – 1.42] | 0.0070 |
| Vilazodone | 2111 | 8 | 2103 | 1.48 [0.74 – 2.97] | 0.2689 |
| Viloxazine | 525 | 4 | 521 | 1.57 [0.58 – 4.22] | 0.3762 |
| Vortioxetine | 1787 | 2 | 1785 | 0.47 [0.12 – 1.87] | 0.2824 |
| Zimeldine | 919 | 0 | 919 |  |  |

*CI confidence interval, ROR reporting odd ratio.*

^a^Adjusted ROR were calculated in adjusted univariate logistic regression analysis, with adjustment for age, gender, drugs used in parkinsonism and drugs associated with parkinsonism.

* Significant signal was defined as adjusted ROR > 1 with α threshold of 0.001, and the number of case being at least 10.

**Supplementary Table 4.f.** Restless legs syndrome.

| Antidepressants | Number of reports  (n = 625,167) | Case  (n = 1339) | Non-case  (n = 623,828) | Adjusted ROR*  [95% CI] | *p-value* |
| --- | --- | --- | --- | --- | --- |
| Tricyclic antidepressants | 103,139 | 88 | 103,051 | 0.36 [0.29 – 0.45] | <.0001 |
| Serotonin reuptake inhibitors* | 291,020 | 553 | 290,467 | 0.82 [0.73 – 0.91] | 0.0003 |
| Monoamine oxidase inhibitors | 7447 | 4 | 7443 | 0.27 [0.10 – 0.72] | 0.0087 |
| “Other” antidepressants | 223,561 | 694 | 222,867 | 1.89 [1.70 – 2.10] | <.0001 |
| Agomelatine | 1653 | 7 | 1646 | 2.11 [1.00 – 4.44] | 0.0493 |
| Amineptine | 926 | 0 | 926 |  |  |
| Amitriptyline | 49,707 | 51 | 49,656 | 0.44 [0.33 – 0.58] | <.0001 |
| Amoxapine | 1269 | 1 | 1268 | 0.41 [0.06 – 2.90] | 0.3701 |
| Bifemelane | 19 | 0 | 19 |  |  |
| Bupropion | 46,160 | 63 | 46,097 | 0.67 [0.52 – 0.86] | 0.0016 |
| Butriptyline | 12 | 0 | 12 |  |  |
| Citalopram | 85,364 | 187 | 85,177 | 0.98 [0.84 – 1.14] | 0.7680 |
| Clomipramine | 8356 | 4 | 8352 | 0.24 [0.09 – 0.64] | 0.0041 |
| Desipramine | 2405 | 2 | 2403 | 0.42 [0.10 – 1.68] | 0.2187 |
| Desvenlafaxine | 5976 | 18 | 5958 | 1.37 [0.86 – 2.18] | 0.1885 |
| Dibenzepin | 229 | 0 | 229 |  |  |
| Dosulepin | 4885 | 3 | 4882 | 0.31 [0.10 – 0.97] | 0.0437 |
| Doxepin | 7838 | 8 | 7830 | 0.49 [0.25 – 0.99] | 0.0453 |
| Duloxetine* | 43,293 | 183 | 43,110 | 1.88 [1.61 – 2.20] | <.0001 |
| Escitalopram | 34,693 | 86 | 34,607 | 1.11 [0.89 – 1.38] | 0.3564 |
| Etoperidone | 19 | 0 | 19 |  |  |
| Fluoxetine | 70,325 | 98 | 70,227 | 0.64 [0.52 – 0.79] | <.0001 |
| Fluvoxamine | 8062 | 11 | 8051 | 0.68 [0.38 – 1.23] | 0.2057 |
| Hypericum perforatum | 2238 | 3 | 2235 | 0.71 [0.23 – 2.20] | 0.5504 |
| Imipramine | 9972 | 10 | 9962 | 0.50 [0.27 – 0.94] | 0.0310 |
| Iprindole | 82 | 0 | 82 |  |  |
| Iproclozide | 0 |  |  |  |  |
| Iproniazide | 105 | 0 | 105 |  |  |
| Isocarboxazid | 162 | 0 | 162 |  |  |
| Lofepramine | 3392 | 0 | 3392 |  |  |
| Maprotiline | 3410 | 1 | 3409 | 0.15 [0.02 – 1.08] | 0.0597 |
| Medifoxamine | 65 | 0 | 65 |  |  |
| Melitracen | 681 | 0 | 681 |  |  |
| Mianserin | 6049 | 11 | 6038 | 0.95 [0.52 – 1.72] | 0.8599 |
| Milnacipran | 2595 | 1 | 2594 | 0.16 [0.02 – 1.12] | 0.0648 |
| Minaprine | 94 | 0 | 94 |  |  |
| Mirtazapine* | 20,941 | 206 | 20,735 | 5.24 [4.51 – 6.10] | <.0001 |
| Moclobemide | 3176 | 3 | 3173 | 0.49 [0.16 – 1.54] | 0.2234 |
| Nefazodone | 5523 | 4 | 5519 | 0.37 [0.14 – 0.99] | 0.0477 |
| Nialamide | 26 | 0 | 26 |  |  |
| Nomifensine | 1311 | 0 | 1311 |  |  |
| Nortriptyline | 8484 | 6 | 8478 | 0.31 [0.14 – 0.68] | 0.0037 |
| Opipramol | 992 | 1 | 991 | 0.52 [0.07 – 3.67] | 0.5088 |
| Oxaflozane | 2 | 0 | 2 |  |  |
| Oxitriptan | 178 | 1 | 177 | 2.69 [0.38 – 19.19] | 0.3251 |
| Paroxetine | 58,491 | 138 | 58,353 | 1.18 [0.99 – 1.41] | 0.0646 |
| Phenelzine | 2364 | 0 | 2364 |  |  |
| Protriptyline | 479 | 1 | 478 | 1.08 [0.15 – 7.71] | 0.9369 |
| Quinupramine | 20 | 0 | 20 |  |  |
| Reboxetine | 1447 | 1 | 1446 | 0.36 [0.05 – 2.57] | 0.3098 |
| Sertraline | 67,840 | 119 | 67,721 | 0.80 [0.66 – 0.97] | 0.0211 |
| Tianeptine | 1888 | 1 | 1887 | 0.26 [0.04 – 1.86] | 0.1794 |
| Toloxatone | 87 | 0 | 87 |  |  |
| Tranylcypromine | 1527 | 1 | 1526 | 0.31 [0.04 – 2.18] | 0.2380 |
| Trazodone | 19,403 | 46 | 19,357 | 1.01 [0.75 – 1.36] | 0.9542 |
| Trimipramine | 2424 | 4 | 2420 | 0.81 [0.30 – 2.15] | 0.6650 |
| Tryptophan | 12,726 | 1 | 12,725 | 0.04 [0.01 – 0.30] | 0.0016 |
| Venlafaxine | 53,563 | 146 | 53,417 | 1.25 [1.06 – 1.49] | 0.0102 |
| Vilazodone* | 2111 | 17 | 2094 | 3.87 [2.40 – 6.26] | <.0001 |
| Viloxazine | 525 | 0 | 525 |  |  |
| Vortioxetine | 1787 | 3 | 1784 | 0.82 [0.26 – 2.55] | 0.7317 |
| Zimeldine | 919 | 0 | 919 |  |  |

*CI confidence interval, ROR reporting odd ratio.*

^a^Adjusted ROR were calculated in adjusted univariate logistic regression analysis, with adjustment for age, gender, drugs used in restless legs syndrome and drugs associated with restless legs syndrome.

* Significant signal was defined as adjusted ROR > 1 with α threshold of 0.001, and the number of case being at least 10.

**Supplementary Table 4.g.** Tardive dyskinesia.

| Antidepressants | Number of reports  (n = 625,167) | Case  (n = 1338) | Non-case  (n = 623,829) | Adjusted ROR*  [95% CI] | *p-value* |
| --- | --- | --- | --- | --- | --- |
| Tricyclic antidepressants | 103,139 | 193 | 102,946 | 0.80 [0.69 – 0.93] | 0.0040 |
| Serotonin reuptake inhibitors* | 291,020 | 679 | 290,341 | 1.21 [1.08 – 1.34] | 0.0006 |
| Monoamine oxidase inhibitors | 7447 | 13 | 7434 | 0.79 [0.46 – 1.37] | 0.3997 |
| “Other” antidepressants | 223,561 | 453 | 223,108 | 0.94 [0.84 – 1.05] | 0.2780 |
| Agomelatine | 1653 | 1 | 1652 | 0.33 [0.05 – 2.34] | 0.2673 |
| Amineptine | 926 | 0 | 926 |  |  |
| Amitriptyline | 49,707 | 70 | 49,637 | 0.60 [0.47 – 0.76] | <.0001 |
| Amoxapine* | 1269 | 12 | 1257 | 4.42 [2.50 – 7.84] | <.0001 |
| Bifemelane | 19 | 0 | 19 |  |  |
| Bupropion | 46,160 | 79 | 46,081 | 0.83 [0.66 – 1.05] | 0.1167 |
| Butriptyline | 12 | 0 | 12 |  |  |
| Citalopram | 85,364 | 199 | 85,165 | 1.07 [0.92 – 1.25] | 0.3560 |
| Clomipramine | 8356 | 14 | 8342 | 0.72 [0.43 – 1.22] | 0.2244 |
| Desipramine | 2405 | 7 | 2398 | 1.24 [0.59 – 2.60] | 0.5765 |
| Desvenlafaxine | 5976 | 17 | 5959 | 1.38 [0.86 – 2.23] | 0.1853 |
| Dibenzepin | 229 | 0 | 229 |  |  |
| Dosulepin | 4885 | 9 | 4876 | 0.88 [0.46 – 1.70] | 0.7080 |
| Doxepin | 7838 | 20 | 7818 | 1.11 [0.72 – 1.73] | 0.6336 |
| Duloxetine | 43,293 | 85 | 43,208 | 0.94 [0.76 – 1.18] | 0.5975 |
| Escitalopram | 34,693 | 91 | 34,602 | 1.18 [0.96 – 1.47] | 0.1204 |
| Etoperidone | 19 | 0 | 19 |  |  |
| Fluoxetine | 70,325 | 175 | 70,150 | 1.22 [1.04 – 1.43] | 0.0165 |
| Fluvoxamine | 8062 | 15 | 8047 | 0.87 [0.52 – 1.44] | 0.5815 |
| Hypericum perforatum | 2238 | 1 | 2237 | 0.25 [0.04 – 1.77] | 0.1650 |
| Imipramine | 9972 | 25 | 9947 | 1.11 [0.75 – 1.65] | 0.5977 |
| Iprindole | 82 | 0 | 82 |  |  |
| Iproclozide | 0 |  |  |  |  |
| Iproniazide | 105 | 0 | 105 |  |  |
| Isocarboxazid | 162 | 1 | 161 | 2.51 [0.35 – 18.01] | 0.3590 |
| Lofepramine | 3392 | 5 | 3387 | 0.70 [0.29 – 1.69] | 0.4259 |
| Maprotiline | 3410 | 3 | 3407 | 0.42 [0.14 – 1.32] | 0.1378 |
| Medifoxamine | 65 | 0 | 65 |  |  |
| Melitracen | 681 | 6 | 675 | 4.85 [2.17 – 10.87] | 0.0001 |
| Mianserin | 6049 | 4 | 6045 | 0.30 [0.11 – 0.81] | 0.0172 |
| Milnacipran | 2595 | 2 | 2593 | 0.38 [0.10 – 1.52] | 0.1721 |
| Minaprine | 94 | 0 | 94 |  |  |
| Mirtazapine | 20,941 | 52 | 20,889 | 1.11 [0.84 – 1.47] | 0.4516 |
| Moclobemide | 3176 | 2 | 3174 | 0.31 [0.08 – 1.23] | 0.0942 |
| Nefazodone | 5523 | 4 | 5519 | 0.36 [0.13 – 0.96] | 0.0407 |
| Nialamide | 26 | 0 | 26 |  |  |
| Nomifensine | 1311 | 0 | 1311 |  |  |
| Nortriptyline | 8484 | 21 | 8463 | 1.03 [0.67 – 1.59] | 0.8946 |
| Opipramol | 992 | 1 | 991 | 0.53 [0.07 – 3.74] | 0.5212 |
| Oxaflozane | 2 | 0 | 2 |  |  |
| Oxitriptan | 178 | 0 | 178 |  |  |
| Paroxetine | 58,491 | 142 | 58,349 | 1.22 [1.02 – 1.45] | 0.0268 |
| Phenelzine | 2364 | 2 | 2362 | 0.37 [0.09 – 1.50] | 0.1641 |
| Protriptyline | 479 | 0 | 479 |  |  |
| Quinupramine | 20 | 0 | 20 |  |  |
| Reboxetine | 1447 | 1 | 1446 | 0.36 [0.05 – 2.53] | 0.3008 |
| Sertraline | 67,840 | 148 | 67,692 | 1.03 [0.87 – 1.22] | 0.7359 |
| Tianeptine | 1888 | 0 | 1888 |  |  |
| Toloxatone | 87 | 0 | 87 |  |  |
| Tranylcypromine | 1527 | 8 | 1519 | 2.20 [1.09 – 4.42] | 0.0270 |
| Trazodone | 19,403 | 61 | 19,342 | 1.31 [1.01 – 1.69] | 0.0410 |
| Trimipramine | 2424 | 7 | 2417 | 1.30 [0.62 – 2.74] | 0.4905 |
| Tryptophan | 12,726 | 2 | 12,724 | 0.09 [0.02 – 0.35] | 0.0006 |
| Venlafaxine* | 53,563 | 152 | 53,411 | 1.35 [1.14 – 1.59] | 0.0006 |
| Vilazodone | 2111 | 6 | 2105 | 1.35 [0.61 – 3.02] | 0.4626 |
| Viloxazine | 525 | 0 | 525 |  |  |
| Vortioxetine | 1787 | 3 | 1784 | 0.87 [0.28 – 2.71] | 0.8127 |
| Zimeldine | 919 | 0 | 919 |  |  |

*CI confidence interval, ROR reporting odd ratio.*

^a^Adjusted ROR were calculated in adjusted univariate logistic regression analysis, with adjustment for age, gender, drugs used in tardive dyskinesia and drugs associated with tardive dyskinesia.

* Significant signal was defined as adjusted ROR > 1 with α threshold of 0.001, and the number of case being at least 10.

**Supplementary Table 4.h.** Tics.

| Antidepressants | Number of reports  (n = 625,167) | Case  (n = 470) | Non-case  (n = 624,697) | Adjusted ROR*  [95% CI] | *p-value* |
| --- | --- | --- | --- | --- | --- |
| Tricyclic antidepressants | 103,139 | 36 | 103,103 | 0.48 [0.34 – 0.67] | <.0001 |
| Serotonin reuptake inhibitors* | 291,020 | 257 | 290,763 | 1.20 [1.00 – 1.44] | 0.0514 |
| Monoamine oxidase inhibitors | 7447 | 1 | 7446 | 0.19 [0.03 – 1.36] | 0.0989 |
| “Other” antidepressants | 223,561 | 176 | 223,385 | 1.17 [0.97 – 1.41] | 0.0987 |
| Agomelatine | 1653 | 2 | 1651 | 1.70 [0.42 – 6.84] | 0.4534 |
| Amineptine | 926 | 0 | 926 |  |  |
| Amitriptyline | 49,707 | 15 | 49,692 | 0.48 [0.29 – 0.81] | 0.0055 |
| Amoxapine | 1269 | 0 | 1269 |  |  |
| Bifemelane | 19 | 0 | 19 |  |  |
| Bupropion | 46,160 | 35 | 46,125 | 0.85 [0.60 – 1.20] | 0.3466 |
| Butriptyline | 12 | 0 | 12 |  |  |
| Citalopram | 85,364 | 63 | 85,301 | 1.00 [0.77 – 1.30] | 0.9932 |
| Clomipramine | 8356 | 6 | 8350 | 0.79 [0.36 – 1.78] | 0.5758 |
| Desipramine | 2405 | 1 | 2404 | 0.40 [0.06 – 2.83] | 0.3568 |
| Desvenlafaxine | 5976 | 1 | 5975 | 0.23 [0.03 – 1.66] | 0.1453 |
| Dibenzepin | 229 | 0 | 229 |  |  |
| Dosulepin | 4885 | 0 | 4885 |  |  |
| Doxepin | 7838 | 3 | 7835 | 0.61 [0.20 – 1.89] | 0.3875 |
| Duloxetine | 43,293 | 33 | 43,260 | 1.26 [0.89 – 1.80] | 0.1967 |
| Escitalopram | 34,693 | 31 | 34,662 | 1.14 [0.79 – 1.64] | 0.4812 |
| Etoperidone | 19 | 0 | 19 |  |  |
| Fluoxetine | 70,325 | 59 | 70,266 | 0.94 [0.72 – 1.24] | 0.6707 |
| Fluvoxamine | 8062 | 9 | 8053 | 1.12 [0.58 – 2.17] | 0.7328 |
| Hypericum perforatum | 2238 | 0 | 2238 |  |  |
| Imipramine | 9972 | 7 | 9965 | 0.76 [0.36 – 1.61] | 0.4743 |
| Iprindole | 82 | 0 | 82 |  |  |
| Iproclozide | 0 |  |  |  |  |
| Iproniazide | 105 | 0 | 105 |  |  |
| Isocarboxazid | 162 | 0 | 162 |  |  |
| Lofepramine | 3392 | 0 | 3392 |  |  |
| Maprotiline | 3410 | 0 | 3410 |  |  |
| Medifoxamine | 65 | 0 | 65 |  |  |
| Melitracen | 681 | 2 | 679 | 6.06 [1.51 – 24.42] | 0.0112 |
| Mianserin | 6049 | 0 | 6049 |  |  |
| Milnacipran | 2595 | 0 | 2595 |  |  |
| Minaprine | 94 | 0 | 94 |  |  |
| Mirtazapine | 20,941 | 7 | 20,934 | 0.50 [0.24 – 1.06] | 0.0716 |
| Moclobemide | 3176 | 0 | 3176 |  |  |
| Nefazodone | 5523 | 2 | 5521 | 0.49 [0.12 – 1.95] | 0.3084 |
| Nialamide | 26 | 0 | 26 |  |  |
| Nomifensine | 1311 | 0 | 1311 |  |  |
| Nortriptyline | 8484 | 2 | 8482 | 0.36 [0.09 – 1.44] | 0.1472 |
| Opipramol | 992 | 0 | 992 |  |  |
| Oxaflozane | 2 | 0 | 2 |  |  |
| Oxitriptan | 178 | 0 | 178 |  |  |
| Paroxetine | 58,491 | 53 | 58,438 | 1.20 [0.90 – 1.59] | 0.2177 |
| Phenelzine | 2364 | 0 | 2364 |  |  |
| Protriptyline | 479 | 0 | 479 |  |  |
| Quinupramine | 20 | 0 | 20 |  |  |
| Reboxetine | 1447 | 0 | 1447 |  |  |
| Sertraline | 67,840 | 73 | 67,767 | 1.33 [1.04 – 1.71] | 0.0252 |
| Tianeptine | 1888 | 0 | 1888 |  |  |
| Toloxatone | 87 | 0 | 87 |  |  |
| Tranylcypromine | 1527 | 1 | 1526 | 0.94 [0.13 – 6.69] | 0.9506 |
| Trazodone | 19,403 | 8 | 19,395 | 0.58 [0.29 – 1.17] | 0.1307 |
| Trimipramine | 2424 | 0 | 2424 |  |  |
| Tryptophan* | 12,726 | 52 | 12,674 | 8.13 [6.06 – 10.92] | <.0001 |
| Venlafaxine | 53,563 | 33 | 53,530 | 0.81 [0.57 – 1.16] | 0.2520 |
| Vilazodone | 2111 | 1 | 2110 | 0.64 [0.09 – 4.54] | 0.6529 |
| Viloxazine | 525 | 0 | 525 |  |  |
| Vortioxetine | 1787 | 3 | 1784 | 2.23 [0.72 – 6.95] | 0.1668 |
| Zimeldine | 919 | 0 | 919 |  |  |

*CI confidence interval, ROR reporting odd ratio.*

^a^Adjusted ROR were calculated in adjusted univariate logistic regression analysis, with adjustment for age, gender, drugs used in tics and drugs associated with tics.

* Significant signal was defined as adjusted ROR > 1 with α threshold of 0.001, and the number of case being at least 10.

**Supplementary Table 4.i.** Tremor.

| Antidepressants | Number of reports  (n = 625,167) | Case  (n = 17,400) | Non-case  (n = 607,767) | Adjusted ROR*  [95% CI] | *p-value* |
| --- | --- | --- | --- | --- | --- |
| Tricyclic antidepressants | 103,139 | 2262 | 100,877 | 0.76 [0.73 – 0.79] | <.0001 |
| Serotonin reuptake inhibitors* | 291,020 | 8893 | 282,127 | 1.20 [1.16 – 1.23] | <.0001 |
| Monoamine oxidase inhibitors | 7447 | 163 | 7284 | 0.77 [0.66 – 0.90] | 0.0013 |
| “Other” antidepressants | 223,561 | 6082 | 217,479 | 0.97 [0.94 – 1.00] | 0.0528 |
| Agomelatine | 1653 | 21 | 1632 | 0.45 [0.29 – 0.69] | 0.0002 |
| Amineptine | 926 | 8 | 918 | 0.31 [0.15 – 0.61] | 0.0008 |
| Amitriptyline | 49,707 | 911 | 48,796 | 0.64 [0.60 – 0.69] | <.0001 |
| Amoxapine | 1269 | 23 | 1246 | 0.65 [0.43 – 0.98] | 0.0399 |
| Bifemelane | 19 | 0 | 19 |  |  |
| Bupropion* | 46,160 | 1700 | 44,460 | 1.35 [1.28 – 1.42] | <.0001 |
| Butriptyline | 12 | 0 | 12 |  |  |
| Citalopram | 85,364 | 2199 | 83,165 | 0.92 [0.88 – 0.96] | 0.0002 |
| Clomipramine* | 8356 | 326 | 8030 | 1.42 [1.27 – 1.59] | <.0001 |
| Desipramine | 2405 | 74 | 2331 | 1.09 [0.86 – 1.37] | 0.4680 |
| Desvenlafaxine | 5976 | 195 | 5781 | 1.17 [1.01 – 1.35] | 0.0365 |
| Dibenzepin | 229 | 11 | 218 | 1.76 [0.96 – 3.23] | 0.0668 |
| Dosulepin | 4885 | 101 | 4784 | 0.75 [0.61 – 0.91] | 0.0039 |
| Doxepin | 7838 | 147 | 7691 | 0.68 [0.57 – 0.80] | <.0001 |
| Duloxetine* | 43,293 | 1372 | 41,921 | 1.16 [1.10 – 1.23] | <.0001 |
| Escitalopram | 34,693 | 940 | 33,753 | 0.97 [0.91 – 1.04] | 0.3474 |
| Etoperidone | 19 | 3 | 16 | 6.88 [2.01 – 23.50] | 0.0021 |
| Fluoxetine | 70,325 | 1932 | 68,393 | 0.96 [0.92 – 1.01] | 0.1330 |
| Fluvoxamine* | 8062 | 379 | 7683 | 1.73 [1.56 – 1.92] | <.0001 |
| Hypericum perforatum | 2238 | 23 | 2215 | 0.36 [0.24 – 0.54] | <.0001 |
| Imipramine | 9972 | 264 | 9708 | 0.95 [0.84 – 1.07] | 0.3811 |
| Iprindole | 82 | 0 | 82 |  |  |
| Iproclozide | 0 |  |  |  |  |
| Iproniazide | 105 | 2 | 103 | 0.69 [0.17 – 2.79] | 0.6018 |
| Isocarboxazid | 162 | 4 | 158 | 0.87 [0.32 – 2.34] | 0.7799 |
| Lofepramine | 3392 | 66 | 3326 | 0.70 [0.55 – 0.89] | 0.0037 |
| Maprotiline | 3410 | 72 | 3338 | 0.75 [0.59 – 0.95] | 0.0157 |
| Medifoxamine | 65 | 2 | 63 | 1.14 [0.28 – 4.65] | 0.8575 |
| Melitracen | 681 | 31 | 650 | 1.71 [1.19 – 2.45] | 0.0036 |
| Mianserin | 6049 | 81 | 5968 | 0.49 [0.39 – 0.61] | <.0001 |
| Milnacipran | 2595 | 65 | 2530 | 0.90 [0.70 – 1.15] | 0.3902 |
| Minaprine | 94 | 2 | 92 | 0.77 [0.19 – 3.14] | 0.7181 |
| Mirtazapine | 20,941 | 406 | 20,535 | 0.71 [0.64 – 0.78] | <.0001 |
| Moclobemide | 3176 | 64 | 3112 | 0.72 [0.56 – 0.93] | 0.0100 |
| Nefazodone | 5523 | 101 | 5422 | 0.64 [0.53 – 0.78] | <.0001 |
| Nialamide | 26 | 0 | 26 |  |  |
| Nomifensine | 1311 | 20 | 1291 | 0.53 [0.34 – 0.83] | 0.0054 |
| Nortriptyline | 8484 | 196 | 8288 | 0.84 [0.73 – 0.96] | 0.0135 |
| Opipramol | 992 | 20 | 972 | 0.72 [0.72 – 1.13] | 0.1509 |
| Oxaflozane | 2 | 0 | 2 |  |  |
| Oxitriptan | 178 | 5 | 173 | 1.00 [0.41 – 2.43] | 0.9963 |
| Paroxetine* | 58,491 | 2334 | 56,157 | 1.52 [1.45 – 1.58] | <.0001 |
| Phenelzine | 2364 | 56 | 2308 | 0.83 [0.64 – 1.08] | 0.1633 |
| Protriptyline | 479 | 11 | 468 | 0.81 [0.45 – 1.47] | 0.4862 |
| Quinupramine | 20 | 1 | 19 | 1.94 [0.26 – 14.27] | 0.5142 |
| Reboxetine | 1447 | 25 | 1422 | 0.62 [0.42 – 0.92] | 0.0169 |
| Sertraline* | 67,840 | 2034 | 65,806 | 1.08 [1.03 – 1.14] | 0.0008 |
| Tianeptine | 1888 | 26 | 1862 | 0.51 [0.35 – 0.75] | 0.0006 |
| Toloxatone | 87 | 1 | 86 | 0.42 [0.06 – 3.04] | 0.3928 |
| Tranylcypromine | 1527 | 36 | 1491 | 0.83 [0.60 – 1.16] | 0.2686 |
| Trazodone | 19,403 | 393 | 19,010 | 0.73 [0.66 – 0.81] | <.0001 |
| Trimipramine | 2424 | 62 | 2362 | 0.93 [0.72 – 1.20] | 0.5772 |
| Tryptophan | 12,726 | 128 | 12,598 | 0.36 [0.30 – 0.43] | <.0001 |
| Venlafaxine | 53,563 | 1595 | 51,968 | 1.07 [1.02 – 1.13] | 0.0111 |
| Vilazodone | 2111 | 82 | 2029 | 1.41 [1.13 – 1.76] | 0.0025 |
| Viloxazine | 525 | 6 | 519 | 0.43 [0.19 – 0.95] | 0.0372 |
| Vortioxetine | 1787 | 30 | 1757 | 0.59 [0.41 – 0.85] | 0.0041 |
| Zimeldine | 919 | 12 | 907 | 0.47 [0.26 – 0.82] | 0.0084 |

*CI confidence interval, ROR reporting odd ratio.*

^a^Adjusted ROR were calculated in adjusted univariate logistic regression analysis, with adjustment for age, gender, drugs used in tremor and drugs associated with tremor.

* Significant signal was defined as adjusted ROR > 1 with α threshold of 0.001, and the number of case being at least 10.

**Supplementary Table 5.** Sensitivity analyses for the antidepressants most frequently reported with the 9 subtypes of movement disorders among 58 antidepressants. For each of these antidepressants, the study period was counted from the year registering its first report in VigiBase^®^ to 1 February, 2017.

| **Antidepressants** | **Number of reports** | **Case** | **Non-case** | **Study period** | **Number of**  **reports of 58 ADs** | **Adjusted ROR***  **[95% CI]** | ***p-value*** |
| --- | --- | --- | --- | --- | --- | --- | --- |
| **Akathisia** | | | | | | | |
| Citalopram* | 85,364 | 249 | 85,115 | 01/01/1967 – 01/02/2017  **01/01/1982 – 01/02/2017** | 625,167  **618,020** | 1.60 [1.39 – 1.84]  **1.59 [1.38 – 1.83]** | <.0001  **<.0001** |
| Escitalopram* | 34,693 | 136 | 34,557 | 01/01/1967 – 01/02/2017  **01/01/2000 – 01/02/2017** | 625,167  **501,127** | 2.02 [1.69 – 2.42]  **1.78 [1.49 – 2.13]** | <.0001  **<.0001** |
| Fluvoxamine* | 8062 | 32 | 8030 | 01/01/1967 – 01/02/2017  **01/01/1983 – 01/02/2017** | 625,167  **616,925** | 1.61 [1.13 – 2.29]  **1.60 [1.12 – 2.27]** | 0.0086  **0.0095** |
| Mianserin* | 6049 | 24 | 6025 | 01/01/1967 – 01/02/2017  **01/01/1976 – 01/02/2017** | 625,167  **622,436** | 2.49 [1.66 – 3.74]  **2.48 [1.65 – 3.73]** | <.0001  **<.0001** |
| Mirtazapine* | 20,941 | 96 | 20,845 | 01/01/1967 – 01/02/2017  **01/01/1996 – 01/02/2017** | 625,167  **541,151** | 2.55 [2.06 – 3.14]  **2.37 [1.92 – 2.93]** | <.0001  **<.0001** |
| Paroxetine* | 58,491 | 166 | 58,325 | 01/01/1967 – 01/02/2017  **01/01/1987 – 01/02/2017** | 625,167  **607,665** | 1.49 [1.27 – 1.76]  **1.49 [1.27 – 1.76]** | <.0001  **<.0001** |
| **Bruxism** | | | | | | | |
| Citalopram* | 85,364 | 133 | 85,231 | 01/01/1967 – 01/02/2017  **01/01/1982 – 01/02/2017** | 625,167  **618,020** | 1.48 [1.22 – 1.78]  **1.46 [1.21 – 1.76]** | <.0001  **<.0001** |
| Desvenlafaxine* | 5976 | 14 | 5962 | 01/01/1967 – 01/02/2017  **01/01/2003 – 01/02/2017** | 625,167  **441,913** | 2.01 [1.18 – 3.41]  **1.69 [1.00 – 2.88]** | 0.0101  **0.0524** |
| Duloxetine* | 43,293 | 82 | 43,211 | 01/01/1967 – 01/02/2017  **01/01/2004 – 01/02/2017** | 625,167  **426,486** | 1.83 [1.46 – 2.31]  **1.55 [1.23 – 1.97]** | <.0001  **0.0002** |
| Escitalopram* | 34,693 | 63 | 34,630 | 01/01/1967 – 01/02/2017  **01/01/2000 – 01/02/2017** | 625,167  **501,127** | 1.62 [1.25 – 2.10]  **1.44 [1.11 – 1.87]** | 0.0003  **0.0063** |
| Paroxetine* | 58,491 | 95 | 58,396 | 01/01/1967 – 01/02/2017  **01/01/1987 – 01/02/2017** | 625,167  **607,665** | 1.43 [1.15 – 1.78]  **1.39 [1.12 – 1.72]** | 0.0011  **0.0029** |
| Sertraline* | 67,840 | 124 | 67,716 | 01/01/1967 – 01/02/2017  **01/01/1991 – 01/02/2017** | 625,167  **587,880** | 1.63 [1.35 – 1.98]  **1.53 [1.26 – 1.85]** | <.0001  **<.0001** |
| Venlafaxine* | 53,563 | 115 | 53,448 | 01/01/1967 – 01/02/2017  **01/01/1994 – 01/02/2017** | 625,167  **569,095** | 1.98 [1.63 – 2.42]  **1.80 [1.47 – 2.20]** | <.0001  **<.0001** |
| Vortioxetine* | 1787 | 10 | 1777 | 01/01/1967 – 01/02/2017  **01/01/2013 – 01/02/2017** | 625,167  **204,206** | 4.71 [2.52 – 8.80]  **4.43 [2.34 – 8.36]** | <.0001  **<.0001** |
| **Dystonia** | | | | | | | |
| Amoxapine* | 1269 | 30 | 1239 | 01/01/1967 – 01/02/2017  **01/01/1981 – 01/02/2017** | 625,167  **618,813** | 4.43 [3.07 – 6.39]  **4.45 [3.08 – 6.41]** | <.0001  **<.0001** |
| Clomipramine* | 8356 | 72 | 8284 | 01/01/1967 – 01/02/2017  **01/01/1969 – 01/02/2017** | 625,167  **625,056** | 1.33 [1.05 – 1.69]  **1.33 [1.05 – 1.69]** | 0.0165  **0.0164** |
| Fluoxetine* | 70,325 | 621 | 69,704 | 01/01/1967 – 01/02/2017  **01/01/1986 – 01/02/2017** | 625,167  **610,019** | 1.51 [1.39 – 1.65]  **1.52 [1.40 – 1.66]** | <.0001  **<.0001** |
| Fluvoxamine* | 8062 | 79 | 7893 | 01/01/1967 – 01/02/2017  **01/01/1983 – 01/02/2017** | 625,167  **616,925** | 1.48 [1.18 – 1.86]  **1.49 [1.19 – 1.86]** | 0.0006  **0.0006** |
| Paroxetine* | 58,491 | 558 | 57,933 | 01/01/1967 – 01/02/2017  **01/01/1987 – 01/02/2017** | 625,167  **607,665** | 1.87 [1.70 – 2.05]  **1.88 [1.72 – 2.07]** | <.0001  **<.0001** |
| **Myoclonus** | | | | | | | |
| Citalopram* | 85,364 | 215 | 85,149 | 01/01/1967 – 01/02/2017  **01/01/1982 – 01/02/2017** | 625,167  **618,020** | 1.31 [1.13 – 1.52]  **1.30 [1.12 – 1.51]** | 0.0003  **0.0005** |
| Clomipramine* | 8356 | 43 | 8313 | 01/01/1967 – 01/02/2017  **01/01/1969 – 01/02/2017** | 625,167  **625,056** | 2.73 [2.01 – 3.71]  **2.73 [2.01 – 3.70]** | <.0001  **<.0001** |
| Fluvoxamine* | 8062 | 28 | 8034 | 01/01/1967 – 01/02/2017  **01/01/1983 – 01/02/2017** | 625,167  **616,925** | 1.81 [1.25 – 2.64]  **1.80 [1.23 – 2.61]** | 0.0019  **0.0023** |
| Mirtazapine* | 20,941 | 69 | 20,872 | 01/01/1967 – 01/02/2017  **01/01/1996 – 01/02/2017** | 625,167  **541,151** | 1.61 [1.26 – 2.06]  **1.56 [1.22 – 1.99]** | 0.0001  **0.0004** |
| Paroxetine* | 58,491 | 155 | 59,336 | 01/01/1967 – 01/02/2017  **01/01/1987 – 01/02/2017** | 625,167  **607,665** | 1.43 [1.21 – 1.69]  **1.41 [1.19 – 1.67]** | <.0001  **<.0001** |
| Phenelzine* | 2364 | 21 | 2343 | 01/01/1967 – 01/02/2017  **01/01/1968 – 01/02/2017** | 625,167  **625,166** | 4.85 [3.14 – 7.49]  **4.85 [3.14 – 7.49]** | <.0001  **<.0001** |
| Venlafaxine* | 53,563 | 132 | 53,431 | 01/01/1967 – 01/02/2017  **01/01/1994 – 01/02/2017** | 625,167  **569,095** | 1.31 [1.09 – 1.57]  **1.26 [1.05 – 1.51]** | 0.0036  **0.0134** |
| **Parkinsonism** | | | | | | | |
| Citalopram* | 85,364 | 361 | 85,003 | 01/01/1967 – 01/02/2017  **01/01/1982 – 01/02/2017** | 625,167  **618,020** | 1.21 [1.08 – 1.36]  **1.20 [1.07 – 1.35]** | 0.0010  **0.0018** |
| Maprotiline* | 3410 | 19 | 3391 | 01/01/1967 – 01/02/2017  **01/01/1975 – 01/02/2017** | 625,167  **622,768** | 1.66 [1.05 – 2.61]  **1.65 [1.05 – 2.60]** | 0.0303  **0.0316** |
| Mirtazapine* | 20,941 | 113 | 20,828 | 01/01/1967 – 01/02/2017  **01/01/1996 – 01/02/2017** | 625,167  **541,151** | 1.24 [1.02 – 1.50]  **1.17 [0.96 – 1.41]** | 0.0296  **0.1175** |
| Paroxetine* | 58,491 | 214 | 58,277 | 01/01/1967 – 01/02/2017  **01/01/1987 – 01/02/2017** | 625,167  **607,665** | 1.16 [1.01 – 1.34]  **1.13 [0.98 – 1.30]** | 0.0424  **0.0918** |
| Sertraline* | 67,840 | 272 | 67,568 | 01/01/1967 – 01/02/2017  **01/01/1991 – 01/02/2017** | 625,167  **587,880** | 1.23 [1.08 – 1.39]  **1.18 [1.04 – 1.34]** | 0.0019  **0.0128** |
| Venlafaxine* | 53,563 | 199 | 53,364 | 01/01/1967 – 01/02/2017  **01/01/1994 – 01/02/2017** | 625,167  **569,095** | 1.22 [1.06 – 1.42]  **1.17 [1.01 – 1.35]** | 0.0070  **0.0422** |
| **Restless legs syndrome** | | | | | | | |
| Duloxetine* | 43,293 | 183 | 43,110 | 01/01/1967 – 01/02/2017  **01/01/2004 – 01/02/2017** | 625,167  **426,486** | 1.88 [1.61 – 2.20]  **1.47 [1.26 – 1.72]** | <.0001  **<.0001** |
| Mirtazapine* | 20,941 | 206 | 20,735 | 01/01/1967 – 01/02/2017  **01/01/1996 – 01/02/2017** | 625,167  **541,151** | 5.24 [4.51 – 6.10]  **4.76 [4.09 – 5.54]** | <.0001  **<.0001** |
| Venlafaxine* | 53,563 | 146 | 53,417 | 01/01/1967 – 01/02/2017  **01/01/1994 – 01/02/2017** | 625,167  **569,095** | 1.25 [1.06 – 1.49]  **1.15 [0.97 – 1.37]** | 0.0102  **0.1043** |
| Vilazodone* | 2111 | 17 | 2094 | 01/01/1967 – 01/02/2017  **01/01/2010 – 01/02/2017** | 625,167  **298,148** | 3.87 [2.40 – 6.26]  **2.94 [1.82 – 4.76]** | <.0001  **<.0001** |
| **Tardive dyskinesia** | | | | | | | |
| Amoxapine* | 1269 | 12 | 1257 | 01/01/1967 – 01/02/2017  **01/01/1981 – 01/02/2017** | 625,167  **618,813** | 4.42 [2.50 – 7.84]  **4.39 [2.48 – 7.78]** | <0.0001  **<.0001** |
| Fluoxetine* | 70,325 | 175 | 70,150 | 01/01/1967 – 01/02/2017  **01/01/1986 – 01/02/2017** | 625,167  **610,019** | 1.22 [1.04 – 1.43]  **1.20 [1.02 – 1.41]** | 0.0165  **0.0260** |
| Paroxetine* | 58,491 | 142 | 58,349 | 01/01/1967 – 01/02/2017  **01/01/1987 – 01/02/2017** | 625,167  **607,665** | 1.22 [1.02 – 1.45]  **1.20 [1.01 – 1.43]** | 0.0268  **0.0383** |
| Trazodone* | 19,403 | 61 | 19,342 | 01/01/1967 – 01/02/2017  **01/01/1981 – 01/02/2017** | 625,167  **618,813** | 1.31 [1.01 – 1.69]  **1.30 [1.00 – 1.68]** | 0.0410  **0.0476** |
| Venlafaxine* | 53,563 | 152 | 53,411 | 01/01/1967 – 01/02/2017  **01/01/1994 – 01/02/2017** | 625,167  **569,095** | 1.35 [1.14 – 1.59]  **1.32 [1.11 – 1.56]** | 0.0006  **0.0016** |
| **Tics** | | | | | | | |
| Sertraline* | 67,840 | 73 | 67,767 | 01/01/1967 – 01/02/2017  **01/01/1991 – 01/02/2017** | 625,167  **587,880** | 1.33 [1.04 – 1.71]  **1.25 [0.97 – 1.60]** | 0.0252  **0.0845** |
| Tryptophan* | 12,726 | 52 | 12,674 | 01/01/1967 – 01/02/2017  **01/01/1973 – 01/02/2017** | 625,167  **607,665** | 8.13 [6.06 – 10.92]  **8.11 [6.04 – 10.88]** | <.0001  **<.0001** |
| **Tremor** | | | | | | | |
| Bupropion* | 46,160 | 1700 | 44,460 | 01/01/1967 – 01/02/2017  **01/01/1985 – 01/02/2017** | 625,167  **611,643** | 1.35 [1.28 – 1.42]  **1.35 [1.28 – 1.42]** | <.0001  **<.0001** |
| Clomipramine* | 8356 | 326 | 8030 | 01/01/1967 – 01/02/2017  **01/01/1969 – 01/02/2017** | 625,167  **625,056** | 1.42 [1.27 – 1.59]  **1.42 [1.27 – 1.59]** | <.0001  **<.0001** |
| Desvenlafaxine* | 5976 | 195 | 5781 | 01/01/1967 – 01/02/2017  **01/01/2003 – 01/02/2017** | 625,167  **441,913** | 1.17 [1.01 – 1.35]  **1.23 [1.07 – 1.42]** | 0.0365  **0.0047** |
| Duloxetine* | 43,293 | 1372 | 41,921 | 01/01/1967 – 01/02/2017  **01/01/2004 – 01/02/2017** | 625,167  **426,486** | 1.16 [1.10 – 1.23]  **1.27 [1.19 – 1.34]** | <.0001  **<.0001** |
| Fluvoxamine* | 8062 | 379 | 7683 | 01/01/1967 – 01/02/2017  **01/01/1983 – 01/02/2017** | 625,167  **616,925** | 1.73 [1.56 – 1.92]  **1.74 [1.56 – 1.93]** | <.0001  **<.0001** |
| Melitracen* | 681 | 31 | 650 | 01/01/1967 – 01/02/2017  **01/01/1975 – 01/02/2017** | 625,167  **622,768** | 1.71 [1.19 – 2.45]  **1.71 [1.20 – 2.46]** | 0.0036  **0.0034** |
| Paroxetine* | 58,491 | 2334 | 56,157 | 01/01/1967 – 01/02/2017  **01/01/1987 – 01/02/2017** | 625,167  **607,665** | 1.52 [1.45 – 1.58]  **1.51 [1.45 – 1.58]** | <.0001  **<.0001** |
| Sertraline* | 67,840 | 2034 | 65,806 | 01/01/1967 – 01/02/2017  **01/01/1991 – 01/02/2017** | 625,167  **587,880** | 1.08 [1.03 – 1.14]  **1.08 [1.03 – 1.14]** | 0.0008  **0.0009** |
| Venlafaxine* | 53,563 | 1595 | 51,968 | 01/01/1967 – 01/02/2017  **01/01/1994 – 01/02/2017** | 625,167  **569,095** | 1.07 [1.02 – 1.13]  **1.07 [1.01 – 1.13]** | 0.0111  **0.0126** |
| Vilazodone* | 2111 | 82 | 2029 | 01/01/1967 – 01/02/2017  **01/01/2010 – 01/02/2017** | 625,167  **298,148** | 1.41 [1.13 – 1.76]  **1.62 [1.30 – 2.03]** | 0.0025  **<.0001** |

*CI confidence interval, ROR reporting odd ratio.*

^a^Adjusted ROR were calculated in adjusted univariate logistic regression analysis, with adjustment for age, gender, drugs associated with movement disorders and drugs used to treat movement disorders.

* Significant signal was defined as adjusted ROR > 1 with α threshold of 0.001, and the number of cases being at least 10.
